# Design, Synthesis and Biological Evaluation of *N*,*N*-Substituted Amine Derivatives as Cholesteryl Ester Transfer Protein Inhibitors

**DOI:** 10.3390/molecules22101658

**Published:** 2017-10-03

**Authors:** Xinran Wang, Lijuan Hao, Xuanqi Xu, Wei Li, Chunchi Liu, Dongmei Zhao, Maosheng Cheng

**Affiliations:** 1 Key Laboratory of Structure-Based Drug Design & Discovery of Ministry of Education, Shenyang Pharmaceutical University, Shenyang 110016, China; wangxinrancai@163.com (X.W.); 18341482606@163.com (L.H.); 15542173071@163.com (W.L.); liuchunchi1990@163.com (C.L.); mscheng@263.net (M.C.); 2 Department of Chemistry, University of Wisconsin-Madison, Madison, WI 53715, USA; xxu53@wisc.edu

**Keywords:** synthesis, *N*,*N*-substituted amine derivatives, CETP inhibitors, HDL-C

## Abstract

*N*,*N*-Substituted amine derivatives were designed by utilizing a bioisosterism strategy. Consequently, twenty-two compounds were synthesized and evaluated for their inhibitory activity against CETP. Structure-activity relationship (SAR) studies indicate that hydrophilic groups at the 2-position of the tetrazole and 3,5-bistrifluoromethyl groups on the benzene ring provide important contributions to the potency. Among these compounds, compound **17** exhibited excellent CETP inhibitory activity (IC_50_ = 0.38 ± 0.08 μM) in vitro. Furthermore, compound **17** was selected for an in vitro metabolic stability study.

## 1. Introduction

Cholesteryl ester transfer protein (CETP) is a hydrophobic glycoprotein secreted predominately by the liver. CETP facilitates the movement of cholesteryl esters (CEs) from high-density lipoprotein (HDL) to low-density lipoprotein (LDL) and very low-density lipoprotein (VLDL), in exchange for triglycerides (TGs) [1,2]. Epidemiological studies have provided compelling evidence to demonstrate an inverse association between HDL-C and cardiovascular events [3,4,5]. Therefore, CETP inhibitors can provide benefits to high risk coronary heart disease (CHD) patients by increasing HDL-C plasma levels [1,6,7,8].

**Figure 1:**
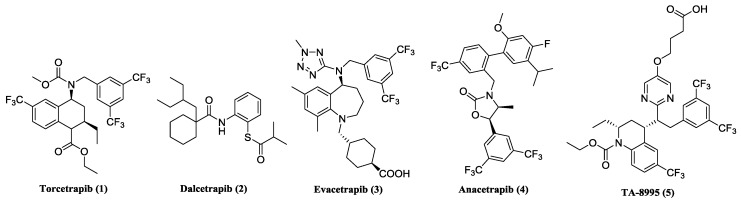
Representative CETP inhibitors.

Torcetrapib (**1**, Figure 1) was the first CETP inhibitor to undergo advanced clinical development. Although early studies have demonstrated that torcetrapib exhibited a significant lipid-regulating ability, subsequent trials indicated that this inhibitor possessed off-target effects affecting blood pressure and aldosterone levels, and failed to demonstrate any favourable impact on CHD patients [9,10,11,12,13]. Accordingly, torcetrapib’s phase III trial was halted in 2006. Dalcetrapib (**2**, Figure 1) was a modest inhibitor without the off-targets effect seen with torcetrapib, elevating HDL-C levels by up to 30% and with a minimal effect on LDL-C levels [14,15]. However, dal-OUTCOMES study was stopped due to lack of clinical benefit [16]. More recently, a Canadian study found that dalcetrapib exhibited potentially clinical effects on atherosclerotic patients with polymorphisms in the ADCY9 gene using a genome-wide approach [17]. Evacetrapib (**3**, Figure 1) possessed profound lipid-regulating effects, raising HDL-C levels by more than 129% and decreasing LDL-C by up to 36%. Evacetrapib did not exhibit torcetrapib-like side-effects, but its phase III ACCELERATE study was terminated due to clinical futility [18,19]. Anacetrapib (**4**, Figure 1) a potent CETP inhibitor currently in phase III, could raise HDL-C levels to 130% and decrease LDL-C levels to 40%. Researchers found that anacetrapib demonstrated potential clinical benefits and would not produce adverse clinical effects similar to those observed with torcetrapib in the DEFINE study [20,21]. TA-8995 (**5**, Figure 1), a novel CETP inhibitor, was well tolerated and had beneficial effects on lipids, raising HDL-C levels by up to 179% and decreasing LDL-C by up to 45% [22,23]. It remains to be seen whether these potent CETP inhibitors will proceed further in the future.

**Figure 2:**
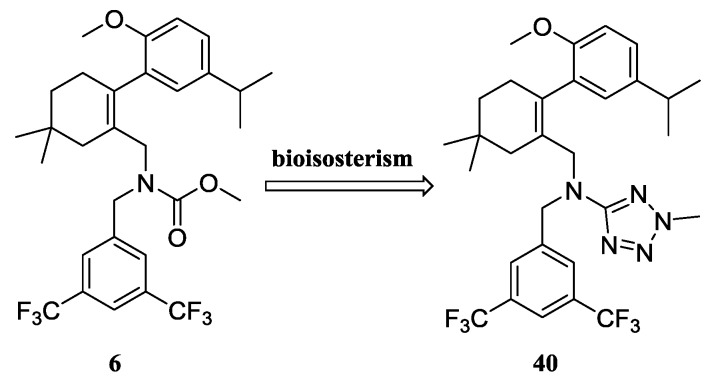
Design of new structures based on compound **6**.

In a previous study, compound **6** (Figure 2) was identified to show weak micromolar activity (IC_50_ = 20.97 μM) [24]. Our initial approach was to build a ring to replace the carbamate part to investigate the effect of steric hindrance on activity. Considering that tetrazole is a bioisoster of the carboxylic acid and the carbamate part of compound **6** could be treated as a carboxylic acid methyl ester, we hold that 2-methyltetrazole is a bioisoster of carbamate. In this paper, our group investigated the cyclization of the carbamate using a bioisosterism strategy to afford a 2-methyltetrazole compound. Fortunately, the compound containing the tetrazole moiety (**40**, Figure 2, IC_50_ = 0.81 ± 0.03 μM) showed increased inhibitory activity. A series of novel *N*,*N*-substituted amine derivatives were then synthesized. Optimization efforts on this scaffold are discussed in this study.

## 2. Results and Discussion

### 2.1. Chemistry

**Scheme 1:**
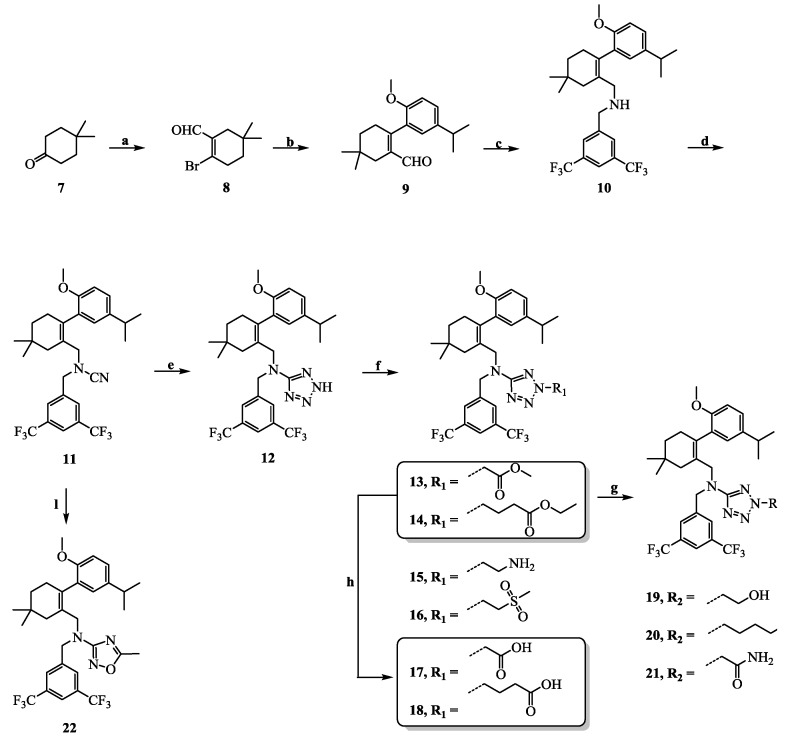
Synthesis of target compounds **12**–**22**. *Reagents and conditions*: (**a**) DMF, PBr_3_, CHCl_3_, rt; (**b**) 5-isopropyl-2-methoxyphenylboronic acid, Pd(OAc)_2_, K_2_CO_3_, acetylacetone, EtOH, 80 °C; (**c**) 3,5-bis(trifluromethyl)benzyl amine, NaBH(OAc)_3_, 1,2-dichloroethane, rt; (**d**) CNBr, DIEA, THF, rt; (**e**) NaN_3_, NH_4_Cl, DMF, 100 °C; (**f**) R_1_-X, Et_3_N, acetonitrile, reflux; or (i) Boc-R_1_-X, Et_3_N, acetonitrile, reflux, (ii) TFA, DCM, rt; or R_1_-OH, PPh_3_, DIAD, rt; (**g**) NaBH_4_/MeOH, THF, reflux; or NH_3_-EtOH, rt; (**h**) (**i**) 1 M aq. NaOH, (ii) 1 M aq. HCl; (**l**) NH_2_OH·HCl, Ac_2_O, Et_3_N, EtOH, reflux.

**Scheme 2:**
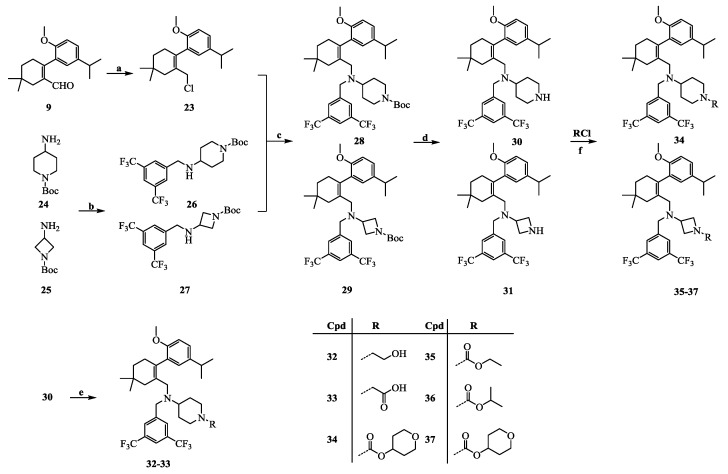
Synthesis of target compounds **32**–**37**. *Reagents and conditions*: (**a**) (i) NaBH_4_, MeOH, rt, (ii) SOCl_2_, DMF, rt; (**b**) 3,5-bis(trifluromethyl)benzaldehyde, NaBH_4_, MeOH, rt; (**c**) NaH, DMF, 0 °C; (**d**) TFA/CH_2_Cl_2_ (1:1), rt; (**e**) (i) methyl 2-bromoacetate, TEA, DMF, rt, (ii) NaBH_4_/MeOH, THF, reflux; or 1 M aq. NaOH, 1 M aq. HCl; (**f**) TEA, DCM, rt.

Compounds **12**–**22** were prepared according to the procedure shown in Scheme 1. The intermediate **10** was obtained in a manner similar to that described in the previously published paper [24]. The treatment of **10** with cyanogen bromide under basic conditions furnished **11** in good yield. Then, the -CN of the resulting intermediate was subjected to cycloaddition with sodium azide to produce **12**. Compound **12** was subjected to substitution reactions with various substituted alkyl bromides to yield compounds **13**–**15**. Alternatively, treatment of **12** with 2-(methylsulfonyl) ethanol under Mitsunobu conditions afforded **16**. Carboxylic acid derivatives **17** and **18** were prepared by hydrolysis of the ester groups of compounds **13** and **14**, respectively. Subsequent reduction or aminolysis reaction of compounds **13** and **14** produced compounds **19**–**21**. Compound **11** reacted with hydroxylamine hydrochloride and subsequently, acetic anhydride, to obtained compound **22**. Compounds **32**–**37** were prepared according to the procedure in Scheme 2. The resulting **9** was reacted with NaBH_4_ and subsequently, SOCl_2_ to afford intermediate **23**. 

**Scheme 3:**
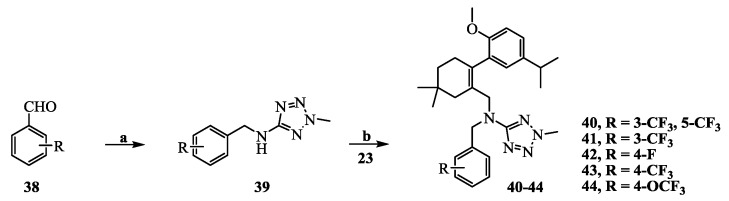
Synthesis of target compounds **40**–**44**. *Reagents and conditions*: (**a**) 2-methyl-2*H*-tetrazol-5-amine, NaBH_4_, MeOH, rt; (**b**) NaH, DMF, 0 °C.

The starting materials **24** and **25** were subjected to reductive amination with 3,5-bis(trifluromethyl)benzaldehyde, which provided **26** and **27**. Intermediates **28** and **29** were obtained by the nucleophilic substitution of **23** by **26** and **27** in the presence of NaH at room temperature. The key intermediates **30** and **31** were obtained by deprotection of 28 and 29 under the condition of TFA/DCM (*v*:*v* = 1:1). Intermediate **30** was reacted with methyl 2-bromoacetate, then the resultant ester intermediate was subjected to reduction conditions to yield compound **32** or hydrolysis conditions to furnish compound **33**. The obtained intermediates **30** and **31** were treated with corresponding chloroformates to give compounds **34**–**37**. As illustrated in Scheme 3, compounds **40**–**44** were synthesized from the corresponding starting benzaldehydes through reductive amination and substitution reactions.

### 2.2. In Vitro Activity Against Cholesteryl Ester Transfer Protein

The *N*,*N*-substituted amine derivatives and the reference compound anacetrapib (**4**) were screened for their in vitro activity against CETP by a BODIPY-CE fluorescence assay with the CETP RP Activity Assay Kit (Catalogue # RB-RPAK; Roar, New York, NY, USA). The results are shown in Table 1. As seen in the table, replacement of a carbamate (**6**, IC_50_ = 20.97 µM) with an *N*-2-methyltetrazole (**40**, IC_50_ = 0.81 ± 0.03 µM) caused a significant increase in the activity. Subsequently, we investigated the relationship between various substituents at the 2-position of the tetrazole (Part A) and the CETP inhibitory activity. The replacement of –CH_3_ by –H (**12**, IC_50_ = 2.84 ± 0.04 µM) was detrimental for activity. Introduction of carboxylic esters (e.g., compounds **13** and **14**) at the 2-position of the tetrazole dramatically reduced the activity, however potency was recovered in the corresponding carboxylic acids (**17** and **18**) and straight chain saturated alcohols (**19** and **20**), where especially two atoms alkyl chains were better. We observed that amine and sulfone groups (**15** and **16**) were tolerated and an amide group (**21**) caused a weak decrease in the activity. Changing *N*-2-methyltetrazole (**40**) to 3-methyoxadiazole (**22**, IC_50_ = 0.72 ± 0.06 µM) showed no advantage. We speculate that carboxylic acids and saturated alcohols at the 2-position of tetrazole provide an important contribution to the potency. 

Next, we investigated the relationship between an aliphatic heterocycle (Part A) and the CETP inhibitory activity. Unfortunately, replacement of *N*-2-substituted tetrazole with azetidine/ piperidine derivatives (compounds **32**–**37**) caused a dramatic decrease in the activity. These results indicate that a flexible fragment in Part A is unfavourable for inhibitory activity.

To further study the relationship between Part B and the CETP inhibitory activity, another five compounds **40**–**44** were prepared and evaluated for their activity. Introduction of trifluoromethyl groups on the 3-position and the 5-position of benzene ring (**40**, IC_50_ = 0.81 ± 0.03 µM) were more beneficial for CETP inhibition. A single trifluoromethyl group on the *meta*-position of the benzene ring (**41**, IC_50_ = 10.05 ± 0.06 µM) caused a severe decrease in the activity. A trifluoromethyl group (**43**), fluorine atom (**42**), and trifluoromethoxy group (**44**) on the *para*-position of the benzene ring resulted in no CETP inhibition.

Half of the compounds showed excellent activities, and four of them exhibited submicromolar activities, but the activity of the most outstanding compound **17** (IC_50_ = 0.38 ± 0.08 μM) was still 10-fold weaker than the reference compound.

### 2.3. In Vitro Metabolic Stability Study

Based on the result of the in vitro CETP inhibitory assay, the potent inhibitor **17** was selected for the in vitro metabolic stability study. As shown in Table 2, compound **17** showed weak stability, with a clearance rate of 119.0 and 146.1 μL/min/mg in human and rat liver microsomes. 

In addition, five cytochrome P450 (CYP) enzymes that commonly metabolize exogenous chemicals were used to test the direct inhibition of compound **17**. Compound **17** possessed favourable metabolic properties, as the inhibition ratios for five CYPs were less than 20% even at the compound concentration of 10 μM.

## 3. Experimental

### 3.1. General Information

All chemicals were obtained from commercial sources and were used without purification unless otherwise specified. Solvents were distilled and dried using standard methods. TLC was performed on silica gel plates with F-254 indicator and visualized by UV-light. The purities of target compounds (all ≥95%). were detected by HPLC, performed on a Waters 1525–2489 system (Waters, Milford, MA, USA). The method conditions were as follows: 100% CH_3_OH or a mixture of solvents H_2_O (A) and MeOH (B) (VA:VB = 5:95) as eluent, flow rate at 1.0 mL/min. Peaks were detected at λ = 254 nm. NMR spectra were recorded on 400 MHz and 600 MHz instruments (Bruker, Karlsruhe, Germany) and the chemical shifts were reported in terms of parts per million with TMS as the internal reference. High-resolution accurate mass determinations (HRMS) for all final target compounds were obtained on a Bruker Micromass Time of Flight mass spectrometer equipped with electrospray ionisation (ESI). Column chromatography was performed with silica gel (200–300 mesh) purchased from Qingdao Haiyang Chemical Co. Ltd. (Qingdao, China)

*N-(3,5-Bis(trifluoromethyl)benzyl)-N-((2-(5-isopropyl-2-methoxyphenyl)-5,5-dimethylcyclohex-1-enyl)-methyl)cyanamide* (**11**): Intermediate **10** (0.3 g, 0.6 mmol) was dissolved in THF (5 mL) and cyanogen bromide (0.2 g, 1.8 mmol) and *N*,*N*-diisopropylethylamine (0.4 mL, 2.4 mmol) were added. The reaction mixture was stirred at room temperature for 5 h and then poured into H_2_O (10 mL) and extracted with EtOAc (10 mL × 3). The combined organic layers were washed with H_2_O (10 mL × 3) and brine (10 mL × 3), dried over Na_2_SO_4_, and concentrated in vacuo. The residue was purified by chromatography on silica gel (petroleum ether:EtOAc = 15:1) to give **11** (0.3 g, 92.8%) as a pale yellow oil. ^1^H-NMR (400 MHz, DMSO-*d*_6_) δ 8.06 (s, 1H), 7.90 (s, 2H), 7.05 (dd, *J* = 8.5, 2.2 Hz, 1H), 6.91–6.77 (m, 2H), 4.29 (s, 2H), 3.64 (s, 3H), 3.49–3.35 (m, 2H), 2.72 (dt, *J* = 13.7, 6.9 Hz, 1H), 2.40–2.28 (m, 1H), 2.11–1.99 (m, 1H), 1.90 (m, 2H), 1.40 (t, *J* = 6.4 Hz, 2H), 1.08 (dd, *J* = 6.9, 1.6 Hz, 6H), 0.98 (d, *J* = 2.8 Hz, 6H). HPLC: *t*_R_ = 13.710 min, 99.27%.

*N-(3,5-Bis(trifluoromethyl)benzyl)-N-((2-(5-isopropyl-2-methoxyphenyl)-5,5-dimethylcyclohex-1-enyl)-methyl)-2H-tetrazol-5-amine* (**12**): Intermediate **11** (0.1 g, 0.2 mmol) was dissolved in DMF (5 mL) and ammonium chloride (0.01 g, 0.8 mmol) and sodium azide (0.05 g, 0.2 mmol) were added. After being stirred at 100 °C for 5 h, the reaction mixture was cooled to room temperature, and H_2_O (20 mL) was added. The mixture was extracted with CH_2_Cl_2_ (20 mL × 3) and the combined organic layers were washed with water (20 mL × 3) and brine (20 mL × 3), dried over Na_2_SO_4_, and concentrated in vacuo. The residue was purified by chromatography on silica gel (petroleum ether:EtOAc = 2:1) to give **12** (0.1 g, 96.3%) as a white solid. Mp 150.6–152.6 °C. ^1^H-NMR (600 MHz, DMSO-*d*_6_) δ 14.88 (s, 1H), 7.94 (s, 1H), 7.66 (s, 2H), 6.99 (dd, *J* = 8.5, 2.2 Hz, 1H), 6.77 (d, *J* = 8.5 Hz, 1H), 6.71 (d, *J* = 2.2 Hz, 1H), 4.55 (s, 2H), 3.91 (m, 2H), 3.61 (s, 3H), 2.61 (dt, *J* = 13.8, 6.9 Hz, 1H), 2.36–2.27 (m, 1H), 2.06–2.03 (m, 1H), 1.77–1.69 (m, 2H), 1.38 (s, 2H), 1.36 (t, *J* = 6.4 Hz, 2H), 1.00 (d, *J* = 6.9 Hz, 6H), 0.91 (d, *J* = 10.3 Hz, 6H). ^13^C-NMR (150 MHz, CDCl_3_) δ: 151.95, 142.58, 139.79, 136.20, 132.01(×2), 129.76, 128.36, 127.92(×2), 127.27, 126.44, 123.95, 122.14, 121.79, 111.11(×2), 55.84(×2), 53.23, 39.75, 34.93, 33.16, 30.00, 28.75, 28.44, 27.24, 24.07, 23.99. HRMS calcd for C_29_H_34_F_6_N_5_O, [M + H]^+^, 582.2589; found 582.2668. HPLC: *t*_R_ = 14.640 min, 96.47%.

*N-(3,5-Bis(trifluoromethyl)benzyl)-N-((2-(5-isopropyl-2-methoxyphenyl)-5,5-dimethylcyclohex-1-enyl)-methyl)-2H-tetrazol-5-amine-2-carboxylic acid methyl ester* (**13**): Compound **12** (0.1 g, 0.2 mmol) and triethylamine (0.1 mL, 0.8 mmol) were dissolved in acetonitrile (2 mL) followed by the addition of methyl 2-bromoacetate (0.03 mL, 0.4 mmol). After being stirred at 80 °C for 2 h, the reaction mixture was cooled to room temperature, and H_2_O (10 mL) was added. The aqueous layer was extracted with EtOAc (5 mL × 3) and the combined organic layers were washed with H_2_O (5 mL × 3) and brine (5 mL × 3), dried over Na_2_SO_4_, and concentrated in vacuo. The residue was purified by chromatography on silica gel (petroleum ether:EtOAc = 4:1) to give **13** (0.09 g, 68.4%) as a colourless oil. ^1^H-NMR (400 MHz, CDCl_3_) δ 7.67 (s, 1H), 7.54 (s, 2H), 7.03 (dd, *J* = 8.4, 2.3 Hz, 1H), 6.76 (d, *J* = 2.3 Hz, 1H), 6.72 (d, *J* = 8.5 Hz, 1H), 5.18 (s, 2H), 4.58–4.38 (m, 2H), 4.19 (d, *J* = 14.5 Hz, 1H), 4.00 (d, *J* = 14.4 Hz, 1H), 3.76 (s, 3H), 3.68 (s, 3H), 2.76 (dt, *J* = 13.8, 6.9 Hz, 1H), 2.54–2.37 (m, 1H), 2.16–2.00 (m, 1H), 1.83 (s, 2H), 1.50–1.33 (m, 2H), 1.15 (d, *J* = 6.9 Hz, 6H), 0.94 (d, *J* = 11.9 Hz, 6H). ^13^C-NMR (150 MHz, CDCl_3_) δ: 169.64, 165.70, 154.12, 141.11, 140.87, 135.34, 131.28(×2), 130.52, 128.06(×2), 127.76(×2), 127.63, 125.66(×2), 110.64(×2), 55.24, 53.01, 52.91, 51.69, 49.38, 40.57, 35.42, 33.03, 29.10, 28.99, 28.03(×2), 23.99(×2). HRMS calcd for C_32_H_38_F_6_N_5_O_3_, [M + H]^+^, 654.2801; found 654.2877. HPLC: *t*_R_ = 8.753 min, 95.8%.

*N-(3,5-Bis(trifluoromethyl)benzyl)-N-((2-(5-isopropyl-2-methoxyphenyl)-5,5-dimethylcyclohex-1-enyl)-methyl)-2H-tetrazol-5-amine-2-butyric acid ethyl ester* (**14**): Colourless oil; yield 71.3%; ^1^H-NMR (400 MHz, CDCl_3_) δ 7.66 (s, 1H), 7.54 (s, 2H), 7.03 (d, *J* = 7.6 Hz, 1H), 6.82–6.65 (m, 2H), 4.46 (d, *J* = 3.5 Hz, 4H), 4.18–4.11(m, 3H), 3.98 (d, *J* = 14.5 Hz, 1H), 3.67 (s, 3H), 2.76 (s, 1H), 2.46 (d, *J* = 19.3 Hz, 1H), 2.30 (d, *J* = 4.5 Hz, 2H), 2.23 (s, 2H), 2.12–2.03 (m, 1H), 1.82 (s, 2H), 1.42 (s, 2H), 1.25–1.24 (m, 3H), 1.14 (s, 6H), 0.94 (d, *J* = 11.1 Hz, 6H). ^13^C-NMR (150 MHz, CDCl_3_) δ: 172.11, 169.37, 154.14, 141.34, 140.85, 135.18, 131.24(×2), 130.57, 128.08, 127.77(×2), 125.63, 124.16, 122.35, 120.60, 110.62(×2), 60.54, 55.22, 51.77, 51.65, 49.36, 40.61, 35.43, 33.03, 30.60, 29.10, 29.00, 28.04, 28.02, 24.12, 23.99, 23.98, 14.04. HRMS calcd for C_35_H_44_F_6_N_5_O_3_, [M + H]^+^, 696.3270; found 656.3361. HPLC: *t*_R_ = 7.653 min, 96.4%.

*2-(2-Aminoethyl)-N-(3,5-Bis(trifluoromethyl)benzyl)-N-((2-(5-isopropyl-2-methoxyphenyl)-5,5-dimethyl-cyclohex-1-enyl)methyl)-2H-tetrazol-5-amine* (**15**): Compound **12** (0.5 g, 0.9 mmol) and triethylamine (1.8 mL, 13.0 mmol) were dissolved in acetonitrile (10 mL), followed by the addition of tert-butyl 2-bromoethylcarbamate (0.6 mL, 2.6 mmol). After being stirred at 80 °C for 2 h, the reaction mixture was cooled to room temperature, and H_2_O (10 mL) was added. The aqueous layer was extracted with EtOAc (5 mL × 3) and the combined organic layers were washed with H_2_O (5 mL × 3) and brine (5 mL × 3), dried over Na_2_SO_4_, and concentrated in vacuo. The residue was immediately dissolved in a trifluoroacetic acid–dichloromethane (1:1) solution (2 mL) and stirred at room temperature overnight. After concentration, the residue was dissolved in EtOAc (5 mL), washed with H_2_O (5 mL × 3) and brine (5 mL × 3), dried over Na_2_SO_4_, and concentrated in vacuo. The residue was purified by chromatography on silica gel (petroleum ether:EtOAc = 2:1) to give **15** (0.35 g, 62.2%) as a colourless oil. ^1^H-NMR (400 MHz, DMSO-*d*_6_) δ 7.93 (s, 1H), 7.66 (s, 2H), 7.00 (dd, *J* = 8.4, 2.2 Hz, 1H), 6.79–6.76 (m, 2H), 4.49 (s, 2H), 4.35 (t, *J* = 6.1 Hz, 2H), 4.02–3.89 (m, 2H), 3.62 (s, 3H), 2.94 (t, *J* = 6.2 Hz, 2H), 2.67 (dt, *J* = 13.8, 6.9 Hz, 1H), 2.54–2.37 (m, 1H), 2.16–2.00 (m, 1H), 1.76 (s, 2H), 1.35 (t, *J* = 6.3 Hz, 2H), 1.04 (d, *J* = 6.9 Hz, 6H), 0.89 (d, *J* = 10.2 Hz, 6H). ^13^C-NMR (150 MHz, CDCl_3_) δ: 169.41, 154.13, 141.31, 140.86, 135.29, 131.25(×2), 130.55, 128.07, 127.80, 127.70, 125.65, 124.14, 122.34, 120.63, 110.62(×2), 55.81, 55.22, 51.72, 49.39, 40.93, 40.64, 35.42, 33.03, 29.11, 29.01, 28.06, 28.03, 24.00, 23.98. HRMS calcd for C_31_H_39_F_6_N_6_O, [M + H]^+^, 625.3011; found 625.3070. HPLC: *t*_R_ = 18.895 min, 96.6%.

*N-(3,5-Bis(trifluoromethyl)benzyl)-N-((2-(5-isopropyl-2-methoxyphenyl)-5,5-dimethylcyclohex-1-enyl)-methyl)-2-(2-(methylsulfonyl)ethyl)-2H-tetrazol-5-amine* (**16**): 2-(methylsulfonyl)ethanol (0.04 mL, 0.4 mmol) was added to a solution of compound **12** (0.1 g, 0.2 mmol) in THF (2 mL) and cooled to 0 °C, followed by the addition of Ph_3_P (0.07 g, 0.3 mmol) and DIAD (0.05 mL, 0.3 mmol). After being stirred at room temperature for 4 h, the reaction mixture was poured into H_2_O (10 mL) and extracted with EtOAc (5 mL × 3), and the combined organic layers were washed with H_2_O (5 mL × 3) and brine (5 mL × 3), dried over Na_2_SO_4_, and concentrated in vacuo. The residue was purified by chromatography on silica gel (petroleum ether:EtOAc = 1:1) to give **16** (0.09 g, 65.3%) as a colourless oil. ^1^H-NMR (400 MHz, CDCl_3_) δ 7.68 (s, 1H), 7.50 (s, 2H), 7.03 (dd, *J* = 8.4, 2.2 Hz, 1H), 6.76 (d, *J* = 2.3 Hz, 1H), 6.71 (d, *J* = 8.5 Hz, 1H), 4.90 (t, *J* = 6.9 Hz, 2H), 4.48 (s, 2H), 4.17 (d, *J* = 15.4 Hz, 1H), 3.99 (d, *J* = 14.4 Hz, 1H), 3.70–3.60 (m, 5H), 2.82–2.71 (m, 4H), 2.46 (d, *J* = 18.1 Hz, 1H), 2.10 (d, *J* = 18.4 Hz, 1H), 1.79 (s, 2H), 1.43 (dd, *J* = 9.7, 6.4 Hz, 2H), 1.15 (d, *J* = 6.9 Hz, 6H), 0.94 (d, *J* = 11.3 Hz, 6H). ^13^C-NMR (150 MHz, CDCl_3_) δ: 169.58, 154.08,140.96, 135.68, 131.36(×2), 130.40, 128.01, 127.69, 127.33, 125.74(×2), 124.10, 122.29, 120.77, 110.66(×2), 55.25, 52.90, 51.66, 49.20, 46.20, 41.37, 40.66, 35.36, 33.03, 29.10, 29.02, 28.08, 28.03, 24.00, 23.98. HRMS calcd for C_32_H_40_F_6_N_5_O_3_S, [M + H]^+^, 688.2678; found 688.2754. HPLC: *t*_R_ = 13.667 min, 95.6%.

*N-(3,5-Bis(trifluoromethyl)benzyl)-N-((2-(5-isopropyl-2-methoxyphenyl)-5,5-dimethylcyclohex-1-enyl)-methyl)-2H-tetrazol-5-amine-2-acetic acid* (**17**): Compound **13** (0.2 g, 0.3 mmol) was dissolved in MeOH (5 mL) and 1 mol/L NaOH (5 mL) was added and the mixture was stirred at room temperature for 2 h. After concentration, the residue was dissolved in H_2_O (10 mL). Then, 1 mol/L HCl (5 mL) was added to the mixture, the mixture was extracted with EtOAc (10 mL × 3), and the combined organic layers were washed with H_2_O (10 mL × 3) and brine (10 mL × 3), dried over Na_2_SO_4_, and concentrated in vacuo. The residue was purified by chromatography on silica gel (DCM:MeOH = 5:1) to give **17** (0.17 g, 86.4%) as a colourless oil. ^1^H-NMR (600 MHz, DMSO-*d*_6_) δ 7.92 (s, 1H), 7.65 (s, 2H), 7.00 (dd, *J* = 8.5, 2.2 Hz, 1H), 6.77 (dd, *J* = 7.9, 5.4 Hz, 2H), 5.35 (s, 2H), 4.57–4.45 (m, 2H), 3.97 (t, *J* = 10.4 Hz, 2H), 3.63 (s, 3H), 2.67 (dt, *J* = 13.7, 6.9 Hz, 1H), 2.33 (d, *J* = 17.9 Hz, 1H), 2.02 (d, *J* = 18.0 Hz, 1H), 1.76 (s, 2H), 1.36 (t, *J* = 6.4 Hz, 2H), 1.04 (d, *J* = 6.9 Hz, 6H), 0.90 (d, *J* = 14.3 Hz, 6H). ^13^C-NMR (150 MHz, DMSO-d_6_) δ: 169.21, 167.87, 154.37, 142.28, 140.54, 134.58, 130.63(×2), 130.29, 127.81, 127.74, 127.62, 125.85, 124.50, 122.69, 120.98, 111.32(×2), 55.70, 53.85, 51.65, 49.55, 40.57, 35.36, 32.75, 29.24, 29.07, 28.46, 28.09, 24.27, 24.21. HRMS calcd for C_31_H_36_F_6_N_5_O_3_, [M + H]^+^, 640.2644; found 640.2709. HPLC: *t*_R_ = 19.013 min, 97.3%.

*N-(3,5-Bis(trifluoromethyl)benzyl)-N-((2-(5-isopropyl-2-methoxyphenyl)-5,5-dimethylcyclohex-1-enyl)-methyl)-2H-tetrazol-5-amine-2-butyric acid* (**18**): Colourless oil; yield 81.3%; ^1^H-NMR (400 MHz, DMSO-*d*_6_) δ 12.18 (s, 1H), 7.90 (s, 1H), 7.65 (s, 2H), 6.99 (d, *J* = 8.5 Hz, 1H), 6.78 (d, *J* = 8.5 Hz, 2H), 4.50 (s, 2H), 4.44 (t, *J* = 6.8 Hz, 2H), 4.00–3.90 (m, 2H), 3.62 (s, 3H), 2.66 (dt, *J* = 13.7, 6.8 Hz, 1H), 2.32 (d, *J* = 17.7 Hz, 1H), 2.19–2.15(m, 2H), 2.07–1.98 (m, 3H), 1.75 (s, 2H), 1.35 (t, *J* = 5.9 Hz, 2H), 1.03 (d, *J* = 6.9 Hz, 6H), 0.88 (d, *J* = 7.5 Hz, 6H). ^13^C-NMR (150 MHz, DMSO-*d*_6_) δ: 173.76, 169.19, 154.39, 142.35, 140.54, 134.46, 130.61(×2), 127.88, 127.74, 127.72(×2), 125.84, 124.51, 122.70, 120.94, 111.33(×2), 55.69, 51.88, 51.70, 49.66, 40.63, 35.36, 32.76, 30.40, 29.25, 29.05, 28.40, 28.13, 24.44, 24.25, 24.21. HRMS calcd for C_33_H_40_F_6_N_5_O_3_, [M + H]^+^, 668.2957; found 668.3050. HPLC: *t*_R_ = 18.767 min, 97.1%.

*N-(3,5-Bis(trifluoromethyl)benzyl)-N-((2-(5-isopropyl-2-methoxyphenyl)-5,5-dimethylcyclohex-1-enyl)-methyl)-2H-tetrazol-5-amine-2-ethyl alcohol* (**19**): Compound **13** (0.4 g, 0.6 mmol) was dissolved in THF (5 mL) and NaBH_4_ (0.05 g, 1.2 mmol) and 2 drops of MeOH were added and the mixture was stirred at 70 °C for 2 h and then cooled to room temperature. After concentration, the residue was dissolved in EtOAc (10 mL), washed with H_2_O (10 mL × 3) and brine (10 mL × 3), dried over Na_2_SO_4_, and concentrated in vacuo. The residue was purified by chromatography on silica gel (petroleum ether:EtOAc = 4:1) to give **19** (0.3 g, 79.8%) as a colourless oil. ^1^H-NMR (600 MHz, DMSO-*d*_6_) δ 7.92 (s, 1H), 7.67 (s, 2H), 7.00 (dd, *J* = 8.5, 2.2 Hz, 1H), 6.78 (d, *J* = 8.5 Hz, 1H), 6.77 (d, *J* = 2.3 Hz, 1H), 4.95 (t, *J* = 5.6 Hz, 1H), 4.49 (s, 2H), 4.42 (t, *J* = 5.4 Hz, 2H), 3.97 (q, *J* = 14.4 Hz, 2H), 3.78 (dd, *J* = 10.8, 5.5 Hz, 2H), 3.62 (s, 3H), 2.67 (dt, *J* = 13.8, 6.9 Hz, 1H), 2.33 (d, *J* = 18.0 Hz, 1H), 2.02 (d, *J* = 18.0 Hz, 1H), 1.76 (s, 2H), 1.35 (t, *J* = 6.4 Hz, 2H), 1.04 (d, *J* = 6.9 Hz, 6H), 0.89 (d, *J* = 16.3 Hz, 6H). ^13^C-NMR (150 MHz, CDCl_3_) δ: 169.30, 154.11, 141.18, 140.89, 135.43, 131.32(×2), 130.51, 128.04, 127.72, 127.55, 125.67, 124.13, 122.32, 120.71, 110.63(×2), 60.30, 55.23, 54.95, 51.80, 49.41, 40.68, 35.40, 33.03, 29.12, 29.02, 28.04(×2), 23.99, 23.98. HRMS calcd for C_31_H_38_F_6_N_5_O_2_, [M + H]^+^, 626.2851; found 626.2920. HPLC: *t*_R_ = 16.776 min, 95.8%.

*N-(3,5-Bis(trifluoromethyl)benzyl)-N-((2-(5-isopropyl-2-methoxyphenyl)-5,5-dimethylcyclohex-1-enyl)-methyl)-2H-tetrazol-5-amine-2-butanol* (**20**): Colourless oil; yield 73.8%; ^1^H-NMR (400 MHz, DMSO-*d*_6_) δ 7.92 (s, 1H), 7.67 (s, 2H), 7.02 (dd, *J* = 8.5, 2.1 Hz, 1H), 6.83–6.76 (m, 2H), 4.52 (s, 2H), 4.48–4.39 (m, 3H), 4.03–3.92 (m, 2H), 3.64 (s, 3H), 3.37 (d, *J* = 5.4 Hz, 2H), 2.68 (dt, *J* = 13.7, 6.8 Hz, 1H), 2.40–2.29 (m, 1H), 2.06–2.00 (m, 1H), 1.89–1.80 (m, 2H), 1.77 (s, 2H), 1.40–1.28 (m, 4H), 1.05 (d, *J* = 6.9 Hz, 6H), 0.90 (d, *J* = 7.5 Hz, 6H). ^13^C-NMR (150 MHz, CDCl_3_) δ: 169.27, 154.14, 141.37, 140.85, 135.19, 131.22(×2), 130.58, 128.09, 127.77(×2), 125.63, 124.16, 122.36, 120.59, 110.62(×2), 61.78, 55.22, 52.52, 51.64, 49.35, 40.60, 35.43, 33.03, 29.12, 29.10, 29.00, 28.04(×2), 25.53, 23.99, 23.98. HRMS calcd for C_33_H_42_F_6_N_5_O_2_, [M + H]^+^, 654.3164; found 654.3257. HPLC: *t*_R_ = 15.755 min, 96.4%.

*2-(5-((3,5-Bis(trifluoromethyl)benzyl)((2-(5-isopropyl-2-methoxyphenyl)-5,5-dimethylcyclohex-1-enyl)-methyl)amino)-2H-tetrazol-2-yl)acetamide* (**21**): Compound **13** (0.2 g, 0.3 mmol) was dissolved in a saturated NH_3_–EtOH solution (5 mL). After being stirred at room temperature for 12 h, the reaction mixture was concentrated in vacuo. The residue was dissolved in EtOAc (10 mL), washed with H_2_O (10 mL × 3) and brine (10 mL × 3), dried over Na_2_SO_4_, and concentrated in vacuo. The residue was purified by chromatography on silica gel (petroleum ether:EtOAc = 4:1) to give **21** (0.12 g, 62.6%) as a colourless oil. ^1^H-NMR (400 MHz, DMSO-*d*_6_) δ 7.91 (s, 1H), 7.70 (s, 1H), 7.66 (s, 2H), 7.42 (s, 1H), 6.99 (dd, *J* = 8.5, 2.2 Hz, 1H), 6.78–6.75(m, 2H), 5.12 (s, 2H), 4.50 (s, 2H), 3.97 (d, *J* = 4.6 Hz, 2H), 3.62 (s, 3H), 2.66 (dt, *J* = 13.7, 6.8 Hz, 1H), 2.33 (d, *J* = 18.0 Hz, 1H), 2.01 (d, *J* = 17.7 Hz, 1H), 1.76 (s, 2H), 1.35 (t, *J* = 6.2 Hz, 2H), 1.04 (d, *J* = 6.9 Hz, 6H), 0.89 (d, *J* = 10.9 Hz, 6H). ^13^C-NMR (150 MHz, CDCl_3_) δ: 169.77, 154.08, 140.96, 140.88, 131.43(×2), 130.42, 127.99(×2), 127.73, 127.29, 125.77(×2), 124.07, 122.26, 120.84, 110.68(×2), 55.24, 54.83, 51.87, 49.43, 40.79, 35.35, 33.04, 29.13, 29.03, 28.10, 28.00, 24.01, 23.98. HRMS calcd for C_31_H_37_F_6_N_6_O_2_, [M + H]^+^, 639.2804; found 639.2909. HPLC: *t*_R_ = 12.757 min, 98.2%.

*N-(3,5-Bis(trifluoromethyl)benzyl)-N-((2-(5-isopropyl-2-methoxyphenyl)-5,5-dimethylcyclohex-1-enyl)-methyl)-5-methyl-1,2,4-oxadiazol-3-amine* (**22**): Intermediate **11** (0.3 g, 0.6 mmol) was dissolved in EtOH (5 mL) and triethylamine (0.08 mL, 0.6 mmol) and hydroxylamine hydrochloride (0.04 g, 0.6 mmol) were added. After being stirred at 80 °C for 2 h, the reaction mixture was cooled to room temperature and concentrated in vacuo. Then, the residue was added pyridine (5 mL) and acetic anhydride (0.07 mL, 0.7 mmol). After being stirred at 80 °C for 12 h, the reaction mixture was cooled to room temperature and concentrated in vacuo. The residue was dissolved in EtOAc (10 mL), washed with H_2_O (10 mL × 3) and brine (10 mL × 3), dried over Na_2_SO_4_, and concentrated in vacuo. The residue was purified by chromatography on silica gel (petroleum ether:EtOAc = 4:1) to give **22** (0.15 g, 41.9%) as a colourless oil. ^1^H-NMR (600 MHz, CDCl_3_) δ 7.66 (s, 1H), 7.50 (s, 2H), 7.00 (dd, *J* = 8.4, 2.1 Hz, 1H), 6.72 (d, *J* = 2.0 Hz, 1H), 6.67 (d, *J* = 8.4 Hz, 1H), 4.43 (q, *J* = 16.2 Hz, 2H), 4.06 (d, *J* = 14.5 Hz, 1H), 3.90 (d, *J* = 14.5 Hz, 1H), 3.65 (s, 3H), 2.73 (dt, *J* = 13.8, 6.9 Hz, 1H), 2.47–2.43 (m, 4H), 2.08 (d, *J* = 18.2 Hz, 1H), 1.81 (s, 2H), 1.42 (dq, *J* = 13.0, 6.5 Hz, 2H), 1.13 (d, *J* = 6.9 Hz, 6H), 0.95 (d, *J* = 13.1 Hz, 6H). ^13^C- NMR (150 MHz, CDCl_3_) δ: 175.39, 170.36, 154.07, 140.92, 140.85, 135.59, 131.31(×2), 130.37, 128.00, 127.54, 127.35, 125.66, 124.13, 122.32, 120.70, 110.62(×2), 55.22, 51.08, 48.61, 40.40, 35.41, 33.01, 29.04, 29.01, 28.10, 27.97, 23.96(×2), 12.63. HRMS calcd for C_31_H_36_F_6_N_3_O_2_, [M + H]^+^, 596.2633; found 596.2716. HPLC: *t*_R_ = 9.953 min, 95.8%.

*2-(2-(Chloromethyl)-4,4-dimethylcyclohex-1-enyl)-4-isopropyl-1-methoxybenzene* (**23**): Intermediate **9** (114.5 mg, 0.4 mmol) was dissolved in ethanol (5 mL). Sodium borohydride (19.0 mg, 0.5 mmol) was added to the mixture. After being stirred at room temperature for 30 min, the reaction mixture was poured into H_2_O (20 mL) and extracted with EtOAc (10 mL × 3). The combined organic layers were washed with H_2_O (10 mL × 3) and brine (10 mL × 3), dried over Na_2_SO_4_, and concentrated in vacuo. SOCl_2_ (0.1 mL, 1.4 mmol) was added to a solution of the above residue in DMF (2 mL) cooled to 0 °C. After being stirred at room temperature for 1 h, the reaction mixture was poured into H_2_O (10 mL) and was extracted with EtOAc (5 mL × 3). The combined organic layers were washed with H_2_O (10 mL × 3) and brine (10 mL × 3), dried over Na_2_SO_4_, and concentrated in vacuo. The residue was purified by chromatography on silica gel (petroleum ether:EtOAc = 10:1) to give **23** (92.5 mg, 75.3% in two steps), which was used immediately for the next step because of its instability. HPLC: *t*_R_ = 7.706 min, 96.8%.

*tert-Butyl 4-(3,5-bis(trifluoromethyl)benzylamino)piperidine-1-carboxylate* (**26**)*:* *tert*-butyl 4-amino-piperidine-1-carboxylate (1.0 g, 5.0 mmol) and 3,5-bis(trifluoromethyl) benzaldehyde (1.0 g, 4.1 mmol) were dissolved in MeOH (15 mL). After stirring for 3 h at room temperature, NaBH_4_ (0.3 g, 7.0 mmol) was added to the mixture. The reaction mixture was stirred at room temperature for 30 min and then poured into a saturated sodium bicarbonate solution (20 mL). The mixture was extracted with CH_2_Cl_2_ (20 mL × 3) and the combined organic layers were washed with water (20 mL × 3) and brine (20 mL × 3), dried over Na_2_SO_4_, and concentrated in vacuo. The residue was purified by chromatography on silica gel (petroleum ether:EtOAc = 4:1) to give **26** (1.4 g, 80.0%) as a pale yellow oil. ^1^H-NMR (600 MHz, CDCl_3_) δ 7.83 (s, 2H), 7.76 (s, 1H), 4.04 (s, 2H), 3.96 (s, 2H), 2.82 (s, 2H), 2.71–2.63 (m, 1H), 1.88 (d, *J* = 11.2 Hz, 2H), 1.46 (s, 9H), 1.35–1.26 (m, 2H). HPLC: *t*_R_ = 13.657 min, 95.7%.

*N-(3,5-Bis(trifluoromethyl)benzyl)-N-((2-(5-isopropyl-2-methoxyphenyl)-5,5-dimethylcyclohex-1-enyl)- methyl)piperidine-4-amine-1-carboxylic acid tert-butyl ester* (**28**): NaH (30.0 mg, 0.7 mmol, 60% in oil) was added to a solution of intermediate **26** (213.1 mg, 0.5 mmol) in DMF (5 mL) cooled to 0 °C. After stirring at 0 °C for 30 min, a solution of intermediate **23** (153.4 mg, 0.5 mmol) in DMF (5 mL) was added. The reaction mixture was allowed to warm to room temperature and stirred for 30 min, and then was poured onto crushed ice. The mixture was diluted with EtOAc (15 mL) and the layers were separated. The aqueous layer was extracted with EtOAc (5 mL × 3), and the combined organic layers were washed with H_2_O (10 mL × 3) and brine (10 mL × 3), dried over Na_2_SO_4_, and concentrated in vacuo. The residue was purified by chromatography on silica gel (petroleum ether:EtOAc = 20:1) to give **28** (235.9 mg, 67.7%) as a colourless oil. ^1^H-NMR (400 MHz, CDCl_3_) δ 7.83 (s, 2H), 7.73 (s, 1H), 7.08 (dd, *J* = 8.4, 2.2 Hz, 1H), 6.79 (d, *J* = 8.4 Hz, 1H), 6.74 (d, *J* = 2.3 Hz, 1H), 4.11 (s, 2H), 3.70 (s, 3H), 3.50 (q, *J* = 15.0 Hz, 2H), 2.90–2.78 (m, 2H), 2.74–2.48 (m, 4H), 2.44–2.31 (m, 1H), 2.09–2.00 (m, 1H), 1.99–1.83 (m, 2H), 1.52 (d, *J* = 12.0 Hz, 1H), 1.45–1.34 (m, 12H), 1.34–1.25 (m, 2H), 1.22 (dd, *J* = 6.9, 1.1 Hz, 6H), 0.96 (s, 6H). HPLC: *t*_R_ = 7.023 min, 96.6%.

*N-(3,5-Bis(trifluoromethyl)benzyl)-N-((2-(5-isopropyl-2-methoxyphenyl)-5,5-dimethylcyclohex-1-enyl)-methyl)piperidin-4-amine* (**30**): Intermediate **28** (0.2 g, 0.3 mmol) was dissolved in a trifluoroacetic acid–dichloromethane (1:1) solution (20 mL) and stirred at room temperature overnight. After concentration, the residue was dissolved in EtOAc (10 mL), washed with H_2_O (10 mL × 3) and brine (10 mL × 3), dried over Na_2_SO_4_, and concentrated in vacuo. The residue was purified by chromatography on silica gel (petroleum ether:EtOAc = 2:1) to give **30** (172.1 mg, 96.1%) as a colourless oil. ^1^H-NMR (400 MHz, DMSO-*d*_6_) δ 8.81 (s, 1H), 8.10–7.88 (m, 3H), 7.11 (dd, *J* = 8.4, 2.1 Hz, 1H), 6.91 (d, *J* = 8.5 Hz, 1H), 6.83 (d, *J* = 2.0 Hz, 1H), 3.66 (s, 3H), 3.57 (d, *J* = 2.4 Hz, 2H), 3.29 (d, *J* = 11.8 Hz, 2H), 2.89–2.68 (m, 6H), 2.26 (d, *J* = 17.6 Hz, 1H), 2.01 (s, 1H), 1.87 (d, *J* = 7.9 Hz, 2H), 1.67–1.48 (m, 4H), 1.35 (t, *J* = 6.2 Hz, 2H), 1.17 (d, *J* = 6.9 Hz, 6H), 0.92 (d, *J* = 10.5 Hz, 6H). HPLC: *t*_R_ = 13.003 min, 95.9%.

*N-(3,5-Bis(trifluoromethyl)benzyl)-N-((2-(5-isopropyl-2-methoxyphenyl)-5,5-dimethylcyclohex-1-enyl)-methyl)piperidin-4-amine-1-ethyl alcohol* (**32**): Intermediate **30** (0.7 g, 1.2 mmol) was dissolved in acetonitrile (10 mL) and methyl 2-bromoacetate (0.4 g, 2.4 mmol) and triethylamine (0.6 mL, 4.7 mmol) were added. The reaction mixture was stirred at room temperature for 16 h and then poured into H_2_O (10 mL) and extracted with EtOAc (10 mL × 3). The combined organic layers were washed with H_2_O (10 mL × 3) and brine (10 mL × 3), dried over Na_2_SO_4_, and concentrated in vacuo. The residue was dissolved in THF (10 mL), and sodium borohydride (0.1 g, 2.6 mmol) and 2 drops of MeOH were added. The mixture was stirred at 70 °C for 2 h and then cooled to room temperature. After concentration, the residue was dissolved in EtOAc (10 mL), washed with H_2_O (10 mL × 3) and brine (10 mL × 3), dried over Na_2_SO_4_, and concentrated in vacuo. The residue was purified by chromatography on silica gel (petroleum ether:EtOAc = 1:1) to give **32** (0.4 g, 52.0%) as a colourless oil. ^1^H-NMR (400 MHz, DMSO-*d*_6_) δ 8.01 (s, 2H), 7.93 (s, 1H), 7.08 (dd, *J* = 8.4, 2.0 Hz, 1H), 6.88 (d, *J* = 8.5 Hz, 1H), 6.74 (d, *J* = 2.0 Hz, 1H), 4.39 (s, 1H), 3.64 (s, 3H), 3.55 (d, *J* = 18.4 Hz, 2H), 3.44 (d, *J* = 5.9 Hz, 2H), 2.84 (d, *J* = 13.3 Hz, 2H), 2.78 (dd, *J* = 13.7, 6.8 Hz, 1H), 2.69 (s, 2H), 2.43–2.37 (m, 3H), 2.26 (d, *J* = 17.8 Hz, 1H), 1.96–1.77 (m, 4H), 1.44–1.42 (m, 7H), 1.16 (d, *J* = 6.9 Hz, 6H), 0.91 (d, *J* = 7.1 Hz, 6H). ^13^C-NMR (150 MHz, CDCl_3_) δ: 154.49, 144.61, 140.59, 132.83, 131.67, 131.25(×2), 129.57, 128.19, 128.00, 125.01, 124.38, 122.57, 120.40, 110.56(×2), 59.29, 57.73, 55.43, 55.13, 53.47, 53.39, 52.55, 52.25, 40.99, 35.60, 33.21(×2), 29.30, 28.93, 28.28, 27.72. 27.25, 24.23, 24.12. HRMS calcd for C_35_H_47_F_6_N_2_O_2_, [M + H]^+^, 641.3463; found 641.3525. HPLC: *t*_R_ = 16.772 min, 97.9%.

*N-(3,5-Bis(trifluoromethyl)benzyl)-N-((2-(5-isopropyl-2-methoxyphenyl)-5,5-dimethylcyclohex-1-enyl)-methyl)piperidin-4-amine-1-acetic acid* (**33**): Intermediate **30** (0.5 g, 0.9 mmol) was dissolved in acetonitrile (10 mL), and methyl 2-bromoacetate (0.3 g, 1.8 mmol) and triethylamine (0.4 mL, 3.4 mmol) were added. The reaction mixture was stirred at room temperature for 16 h and then poured into H_2_O (10 mL) and extracted with EtOAc (10 mL × 3). The combined organic layers were washed with H_2_O (10 mL × 3) and brine (10 mL × 3), dried over Na_2_SO_4_, and concentrated in vacuo. The residue was dissolved in MeOH (5 mL) and 1 mol/L NaOH (5 mL) was added and the mixture was stirred at room temperature for 2 h. After concentration, the residue was dissolved in H_2_O (10 mL). Then, 1 mol/L HCl (5 mL) was added to the mixture, the mixture was extracted with EtOAc (10 mL × 3), and the combined organic layers were washed with H_2_O (10 mL × 3) and brine (10 mL × 3), dried over Na_2_SO_4_, and concentrated in vacuo. The residue was purified by chromatography on silica gel (DCM:MeOH = 5:1) to give **33** (0.4 g, 67.8%) as a colourless oil. ^1^H-NMR (400 MHz, DMSO-*d*_6_) δ 8.02 (s, 2H), 7.95 (s, 1H), 7.09 (dd, *J* = 8.5, 2.1 Hz, 1H), 6.89 (d, *J* = 8.5 Hz, 1H), 6.77 (d, *J* = 2.1 Hz, 1H), 3.65 (s, 3H), 3.63–3.51 (m, 2H), 3.20 (s, 2H), 3.14 (d, *J* = 10.4 Hz, 2H), 2.79 (dt, *J* = 13.7, 6.9 Hz, 1H), 2.70 (s, 2H), 2.62–2.51 (m, 3H), 2.26 (d, *J* = 17.7 Hz, 1H), 1.96 (s, 1H), 1.91–1.75 (m, 2H), 1.70–1.51 (m, 2H), 1.40 (s, 2H), 1.35 (t, *J* = 6.3 Hz, 2H), 1.16 (d, *J* = 6.8 Hz, 6H), 0.91 (d, *J* = 8.7 Hz, 6H). ^13^C-NMR (150 MHz, DMSO-*d*_6_) δ: 168.60, 154.62(×2), 145.76, 140.43, 132.53, 131.21, 130.22(×2), 129.42, 128.93, 127.74, 125.54, 124.78, 122.97, 111.48(×2), 59.03, 55.81, 54.09, 52.70, 52.43, 52.37, 51.58, 41.02, 35.50, 32.94, 29.40, 29.00, 28.53, 27.95, 24.55(×2), 24.44(×2). HRMS calcd for C_35_H_45_F_6_N_2_O_3_, [M + H]^+^, 655.3256; found 655.3345. HPLC: *t*_R_ = 19.973 min, 98.1%.

*N-(3,5-Bis(trifluoromethyl)benzyl)-N-((2-(5-isopropyl-2-methoxyphenyl)-5,5-dimethylcyclohex-1-enyl)-methyl)piperidin-4-amine-1- methanoic acid tetrahydro-2H-pyran-4-ol ester* (**34**): Intermediate **30** (0.2 g, 0.3 mmol) was dissolved in CH_2_Cl_2_ (5 mL), and tetrahydro-2H-pyran-4-yl carbonochloridate (0.1 g, 0.5 mmol) and triethylamine (0.2 mL, 1.4 mmol) were added. The reaction mixture was stirred at room temperature for 30 min and then poured into H_2_O (10 mL) and extracted with CH_2_Cl_2_ (10 mL × 3). The combined organic layers were washed with H_2_O (10 mL × 3) and brine (10 mL × 3), dried over Na_2_SO_4_, and concentrated in vacuo. The residue was purified by chromatography on silica gel (petroleum ether:EtOAc = 4:1) to give **34** (0.1 g, 45.9%) as a colourless oil. ^1^H-NMR (600 MHz, CDCl_3_) δ 7.82 (s, 2H), 7.73 (s, 1H), 7.08 (dd, *J* = 8.3, 2.0 Hz, 1H), 6.79 (d, *J* = 8.4 Hz, 1H), 6.73 (d, *J* = 2.0 Hz, 1H), 4.86–4.80 (m, 1H), 4.13 (d, *J* = 7.1 Hz, 2H), 3.90–3.85 (m, 2H), 3.71 (s, 3H), 3.56–3.53 (m, 3H), 3.49–3.41 (m, 1H), 2.84–2.81 (m, 2H), 2.75–2.53 (m, 4H), 2.37 (d, *J* = 17.7 Hz, 1H), 2.02 (s, 1H), 1.93–1.90 (m, 3H), 1.87 (s, 1H), 1.65–1.63 (m, 3H), 1.55 (d, *J* = 12.5 Hz, 1H), 1.46–1.37 (m, 3H), 1.35–1.29 (m, 1H), 1.22 (dd, *J* = 6.9, 2.4 Hz, 6H), 0.96 (s, 6H). ^13^C-NMR (150 MHz, CDCl_3_) δ: 154.47(×2), 144.35, 140.64, 131.57, 131.32, 131.10, 129.35, 128.18(×2), 125.09(×2), 124.35, 122.54, 120.51, 110.61(×2), 69.63, 65.30(×2), 55.44(×2), 55.35, 52.46, 52.23, 43.82, 43.66, 41.07, 35.57, 33.19, 32.08(×2), 29.24, 28.94, 28.33, 27.64, 24.24, 24.11(×2). HRMS calcd for C_39_H_51_F_6_N_2_O_4_, [M + H]^+^, 725.3675; found 725.3751. HPLC: *t*_R_ = 15.553 min, 96.0%.

*N-(3,5-Bis(trifluoromethyl)benzyl)-N-((5′-isopropyl-2′-methoxy-4,4-dimethyl-3,4,5,6-tetrahydro-[1,1′-biphenyl]-2-yl)methyl)azetidin-3-amine-1-methanoic acid ethyl ester* (**35**): Intermediate **31** (0.2 g, 0.3 mmol) was dissolved in CH2Cl2 (5 mL), and ethyl chloroformate (0.1 g, 0.7 mmol) and triethylamine (0.2 mL, 1.4 mmol) were added. The reaction mixture was stirred at room temperature for 30 min and then poured into H_2_O (10 mL) and extracted with CH_2_Cl_2_ (10 mL × 3). The combined organic layers were washed with H_2_O (10 mL × 3) and brine (10 mL × 3), dried over Na_2_SO_4_, and concentrated in vacuo. The residue was purified by chromatography on silica gel (petroleum ether:EtOAc = 4:1) to give **35** (0.2 g, 89.2%) as a colourless oil. ^1^H-NMR (400 MHz, CDCl_3_) δ 7.78 (s, 2H), 7.76 (s, 1H), 7.09 (dd, *J* = 8.4, 2.1 Hz, 1H), 6.80 (d, *J* = 8.4 Hz, 1H), 6.74 (d, *J* = 2.1 Hz, 1H), 4.07 (q, *J* = 7.1 Hz, 2H), 3.88–3.81 (m, 1H), 3.80–3.72 (m, 3H), 3.71 (s, 3H), 3.68–3.56 (m, 2H), 3.55–3.47 (m, 1H), 2.90–2.77 (m, 2H), 2.77–2.65 (m, 1H), 2.43–2.29 (m, 1H), 2.10–2.04 (m, 1H), 2.01–1.82 (m, 2H), 1.45–1.35 (m, 2H), 1.24–1.19 (m, 9H), 0.96 (s, 6H). ^13^C-NMR (150 MHz, CDCl_3_) δ: 156.60(×2), 154.30(×2), 140.75, 131.29(×2), 131.14, 128.37, 127.94(×2), 125.39, 124.21, 122.41, 120.87, 110.55(×2), 60.92, 55.32, 54.44, 53.43, 41.59, 35.49, 33.14(×2), 29.38, 28.97, 28.22, 27.80, 24.18, 24.12(×2), 14.60(×2). HRMS calcd for C_34_H_43_F_6_N_2_O_3_, [M + H]^+^, 641.3100; found 641.3180. HPLC: *t*_R_ = 11.763 min, 96.7%.

*N-(3,5-Bis(trifluoromethyl)benzyl)-N-((5′-isopropyl-2′-methoxy-4,4-dimethyl-3,4,5,6-tetrahydro-[1,1′-biphenyl]-2-yl)methyl)azetidin-3-amine-1-methanoic acid isopropyl ester* (**36**): Colourless oil; yield 69.3%; ^1^H-NMR (400 MHz, CDCl_3_) δ 7.77 (s, 2H), 7.76 (s, 1H), 7.09 (dd, *J* = 8.4, 2.2 Hz, 1H), 6.80 (d, *J* = 8.4 Hz, 1H), 6.74 (d, *J* = 2.2 Hz, 1H), 4.84 (dt, *J* = 12.5, 6.2 Hz, 1H), 3.83 (t, *J* = 8.4 Hz, 1H), 3.77 (d, *J* = 7.9 Hz, 1H), 3.73 (d, *J* = 5.4 Hz, 2H), 3.70 (s, 3H), 3.67–3.56 (m, 2H), 3.55–3.47 (m, 1H), 2.89–2.77 (m, 2H), 2.76–2.64 (m, 1H), 2.40–2.28 (m, 1H), 2.09–2.04 (m, 1H), 2.00–1.83 (m, 2H), 1.44–1.35 (m, 2H), 1.22 (d, *J* = 6.9 Hz, 6H), 1.19 (d, *J* = 6.2 Hz, 6H), 0.96 (d, *J* = 2.8 Hz, 6H). ^13^C-NMR (150 MHz, CDCl_3_) δ: 156.38(×2), 154.32(×2), 140.72, 131.48, 131.26, 131.19, 128.34, 127.93(×2), 125.35, 124.23, 122.43, 120.83, 110.53(×2), 68.17, 55.31(×2), 54.44, 53.43, 51.10, 41.56, 35.50, 33.14, 29.38, 28.96(×2), 28.23, 27.79, 24.19, 24.13, 22.11(×2). HRMS calcd for C_35_H_45_F_6_N_2_O_3_, [M + H]^+^, 655.3256; found 655.3344. HPLC: *t*_R_ = 10.757 min, 96.8%.

*N-(3,5-Bis(trifluoromethyl)benzyl)-N-((5′-isopropyl-2′-methoxy-4,4-dimethyl-3,4,5,6-tetrahydro-[1,1′-biphenyl]-2-yl)methyl)azetidin-3-amine-1-methanoic acid tetrahydro-2H-pyran-4-ol ester* (**37**): Colourless oil; yield 73.6%; ^1^H NMR (600 MHz, CDCl_3_) δ 7.78 (s, 2H), 7.76 (s, 1H), 7.09 (dd, *J* = 8.4, 2.1 Hz, 1H), 6.80 (d, *J* = 8.4 Hz, 1H), 6.75 (d, *J* = 2.2 Hz, 1H), 4.79 (tt, *J* = 8.4, 4.0 Hz, 1H), 3.89–3.83 (m, 3H), 3.82–3.78 (m, 1H), 3.75 (s, 2H), 3.71 (s, 3H), 3.62 (s, 2H), 3.55–3.49 (m, 3H), 2.90–2.77 (m, 2H), 2.73 (s, 1H), 2.39–2.30 (m, 1H), 2.09–2.02 (m, 1H), 2.00–1.94 (m, 1H), 1.92–1.85 (m, 3H), 1.64–1.59 (m, 2H), 1.45–1.34 (m, 2H), 1.22 (d, *J* = 6.9 Hz, 6H), 0.97 (d, *J* = 4.2 Hz, 6H). ^13^C-NMR (150 MHz, CDCl_3_) δ: 155.77(×2), 154.30, 140.76, 131.53, 131.31, 131.14, 128.36, 127.90(×3), 125.40, 124.20, 122.40, 120.92, 110.57(×2), 69.44, 65.26(×2), 55.33(×2), 54.49, 53.46, 41.59, 35.48, 33.14(×2), 32.07(×2), 29.40, 28.98(×2), 28.23, 27.82, 24.20, 24.13. HRMS calcd for C_37_H_47_F_6_N_2_O_4_, [M + H]^+^, 697.3362; found 697.3459. HPLC: *t*_R_ = 16.003 min, 95.8%.

*N-(3,5-Bis(trifluoromethyl)benzyl)-N-((2-(5-isopropyl-2-methoxyphenyl)-5,5-dimethylcyclohex-1-enyl)-methyl)-2-methyl-2H-tetrazol-5-amine* (**40**): The title compound was obtained in a manner similar to that described for the preparation of intermediate **28**. Colourless oil; yield 70.5%; ^1^H-NMR (600 MHz, DMSO-*d*_6_) δ 7.92 (s, 1H), 7.64 (s, 2H), 6.98 (dd, *J* = 8.4, 2.2 Hz, 1H), 6.77–6.73 (m, 2H), 4.50 (s, 2H), 4.10 (s, 3H), 3.99–3.93 (m, 2H), 3.60 (s, 3H), 2.65 (dt, *J* = 13.7, 6.9 Hz, 1H), 2.32 (d, *J* = 18.0 Hz, 1H), 2.01 (d, *J* = 18.0 Hz, 1H), 1.79–1.72 (m, 2H), 1.35 (t, *J* = 6.4 Hz, 2H), 1.02 (d, *J* = 6.9 Hz, 6H), 0.89 (d, *J* = 13.2 Hz, 6H). ^13^C-NMR (150 MHz, CDCl_3_) δ: 169.46, 154.12, 141.34, 140.83, 135.26, 131.26(×2), 130.54, 128.05, 127.70, 127.65, 125.61, 124.16, 122.35, 120.60, 110.59(×2), 55.21, 51.59, 49.22, 40.56, 39.20, 35.44, 33.02, 29.09, 29.02, 28.08, 27.99, 23.98(×2). HRMS calcd for C_30_H_36_F_6_N_5_O, [M + H]^+^, 596.2746; found 596.2847. HPLC: *t*_R_ = 8.776 min, 96.1%.

*N-((2-(5-Isopropyl-2-methoxyphenyl)-5,5-dimethylcyclohex-1-enyl)methyl)-2-methyl-N-(3-(trifluoromethyl)-benzyl)-2H-tetrazol-5-amine* (**41**): Colourless oil; yield 78.4%; ^1^H-NMR (600 MHz, DMSO-*d*_6_) δ 7.52 (d, *J* = 7.7 Hz, 1H), 7.41 (t, *J* = 7.7 Hz, 1H), 7.34 (s, 1H), 7.22 (d, *J* = 7.8 Hz, 1H), 7.02 (dd, *J* = 8.5, 2.3 Hz, 1H), 6.80 (d, *J* = 8.5 Hz, 1H), 6.77 (d, *J* = 2.3 Hz, 1H), 4.41 (s, 2H), 4.10 (s, 3H), 3.92 (q, *J* = 14.7 Hz, 2H), 3.63 (s, 3H), 2.68 (dt, *J* = 13.8, 6.9 Hz, 1H), 2.32 (d, *J* = 18.0 Hz, 1H), 2.04 (d, *J* = 18.1 Hz, 1H), 1.76 (s, 2H), 1.38 (t, *J* = 6.5 Hz, 2H), 1.05 (dd, *J* = 6.9, 0.8 Hz, 6H), 0.91 (d, *J* = 5.1 Hz, 6H). ^13^C-NMR (150 MHz, CDCl_3_) δ: 169.66, 154.26, 140.64, 139.40, 134.35, 130.80, 130.73, 128.32, 128.03, 125.36, 124.31, 124.29, 123.43, 123.40, 110.45(×2), 55.17, 51.16, 49.31, 40.42, 39.15, 35.54, 33.05, 29.19, 29.03, 28.20, 27.98, 24.09, 24.01. HRMS calcd for C_29_H_37_F_3_N_5_O, [M + H]^+^, 528.2872; found 528.2960. HPLC: *t*_R_ = 8.335 min, 96.2%.

*N-(4-Fluorobenzyl)-N-((2-(5-isopropyl-2-methoxyphenyl)-5,5-dimethylcyclohex-1-enyl)methyl)-2-methyl-2H-tetrazol-5-amine* (**42**): Colourless oil; yield 80.1%; ^1^H-NMR (600 MHz, DMSO-*d*_6_) δ 7.07–7.05 (m, 1H), 6.97–6.93 (m, 4H), 6.86–6.85 (m, 1H), 6.80 (d, *J* = 2.3 Hz, 1H), 4.30 (q, *J* = 15.8 Hz, 2H), 4.10 (s, 3H), 3.85 (dd, *J* = 44.8, 14.8 Hz, 2H), 3.66 (s, 3H), 2.72 (dt, *J* = 13.8, 6.9 Hz, 1H), 2.32 (d, *J* = 18.0 Hz, 1H), 2.08 (d, *J* = 18.0 Hz, 1H), 1.76 (s, 2H), 1.40 (t, *J* = 6.5 Hz, 2H), 1.09 (dd, *J* = 6.9, 0.6 Hz, 6H), 0.93 (s, 6H). ^13^C- NMR (150 MHz, CDCl_3_) δ: 169.67, 154.42, 140.66, 133.58, 130.93, 129.47, 129.41, 128.30, 128.14, 127.90, 125.25, 114.72, 114.58, 110.44(×2), 55.23, 50.53, 48.79, 40.35, 39.12, 35.64, 33.12, 29.28, 29.04, 28.25, 28.01, 24.20, 24.06. HRMS calcd for C_28_H_37_FN_5_O, [M + H]^+^, 478.2904; found 478.2999. HPLC: *t*_R_ = 7.750 min, 96.7%.

*N-((2-(5-Isopropyl-2-methoxyphenyl)-5,5-dimethylcyclohex-1-enyl)methyl)-2-methyl-N-(4-(trifluoromethyl)-benzyl)-2H-tetrazol-5-amine* (**43**): Colourless oil; yield 67.5%; ^1^H-NMR (600 MHz, CDCl_3_) δ 7.37 (d, *J* = 8.1 Hz, 2H), 7.10 (d, *J* = 8.0 Hz, 2H), 7.02 (dd, *J* = 8.4, 2.3 Hz, 1H), 6.76 (d, *J* = 2.3 Hz, 1H), 6.70 (d, *J* = 8.4 Hz, 1H), 4.45 (s, 2H), 4.11 (s, 3H), 4.02 (s, 2H), 3.65 (s, 3H), 2.73 (dt, *J* = 13.8, 6.9 Hz, 1H), 2.44 (d, *J* = 18.2 Hz, 1H), 2.12 (d, *J* = 18.1 Hz, 1H), 1.84 (d, *J* = 1.6 Hz, 2H), 1.48–1.42 (m, 2H), 1.14 (dd, *J* = 6.9, 1.8 Hz, 6H), 0.96 (s, 6H). ^13^C-NMR (150 MHz, CDCl_3_) δ: 169.62, 154.32, 142.35, 140.67, 134.24, 130.74, 128.03, 127.98, 127.70(×3), 125.30, 124.87, 124.85, 110.43(×2), 55.20, 50.82, 49.03, 40.41, 39.16, 35.60, 33.06, 29.22, 29.05, 28.30, 27.93, 24.15, 23.98. HRMS calcd for C_29_H_37_F_3_N_5_O, [M + H]^+^, 528.2872; found 528.2953. HPLC: *t*_R_ = 8.985 min, 96.7%.

*N-((2-(5-Isopropyl-2-methoxyphenyl)-5,5-dimethylcyclohex-1-enyl)methyl)-2-methyl-N-(4-(trifluoro-methoxy)benzyl)-2H-tetrazol-5-amine* (**44**): Colourless oil; yield 66.3%; ^1^H-NMR (600 MHz, DMSO-*d*_6_) δ 7.14 (d, *J* = 8.1 Hz, 2H), 7.06–7.01 (m, 3H), 6.82 (d, *J* = 8.5 Hz, 1H), 6.78 (d, *J* = 2.2 Hz, 1H), 4.35 (s, 2H), 4.11 (s, 3H), 3.88 (m, 2H), 3.66 (s, 3H), 2.70 (dt, *J* = 13.8, 6.9 Hz, 1H), 2.33 (d, *J* = 18.4 Hz, 1H), 2.06 (d, *J* = 18.0 Hz, 1H), 1.76 (s, 2H), 1.39 (t, *J* = 6.4 Hz, 2H), 1.06 (dd, *J* = 6.9, 1.0 Hz, 6H), 0.92 (d, *J* = 1.6 Hz, 6H). ^13^C-NMR (150 MHz, CDCl_3_) δ: 169.66, 154.37, 140.66, 136.94, 133.96, 130.81, 129.00(×3), 128.15, 128.11, 125.29, 120.46(×2), 110.43(×2), 55.19, 50.66, 48.76, 40.39, 39.13, 35.61, 33.10, 29.21, 29.03, 28.25, 27.97, 24.14, 24.01. HRMS calcd for C_29_H_37_F_3_N_5_O_2_, [M + H]^+^, 544.2821; found 544.2898. HPLC: *t*_R_ = 8.302 min, 97.3%.

### 3.2. In Vitro Test for CETP Inhibitory Activity

All tested compounds were dissolved in 100% DMSO, making sure the compound was dissolved totally. The solution was vibrated hard on an oscillator for more than 30 s and then stored in a nitrogen cabinet. The stock solutions (10 mM) were diluted with DMSO for an 8-point titration (1:5 serial dilutions) in a 96-well dilution plate. The activity was estimated in accordance with the instruction for the CETP inhibitor screening kit and recombinant CETP. Compounds were tested at eight concentrations, and the fluorescence intensity were measured using a fluorometer (ExEm = 465/535 nm). The IC_50_ was determined from a curve fit of the data with each concentration tested three times.

### 3.3. Cytochrome P450 Inhibition Assay

Five specific probe substrates (CYP3A4, 2 μM midazolam; CYP2D6, 5 μM dextromethorphan; CYP2C9, 5 μM diclofenac; CYP1A2, 10 μM phenacetin; CYP2C19, 30 μM S-mephenytoin) were used to evaluated cytochrome P450 inhibition in human liver microsomes (0.25 mg/mL). A mixture of 20 μL specific probe substrate solution and 20 μL buffer solution (100 mM K_3_PO_4_, 33 mM MgCl_2_, pH = 7.4) was added compound (2 μL) and 158 μL human liver microsomes solution (0.25 mg/mL). The mixture were incubated at 37 °C for 10 min and another 10 min underwent after 20 μL NADPH solution added. Consecutively, 400 µL cold stop solution (200 ng/mL tolbutamide and 200 ng/mL labetalol in acetonitrile) was added to terminated the reaction. After the reactions were terminated, The plates were centrifuged (4000 rpm) at room temperature for 20 min, and the supernatants were analysed by LC/MS/MS.

### 3.4. Metabolic Stability Study

Ten μL (100 μM/L) of compound and 80 μL liver microsomes were mixture and incubated at 37 °C for 10 min, and then 10 μL NADPH regenerating system was added. Samples were obtained at 0 min, 5 min, 10 min, 20 min, 30 min and 60 min respectively, and 300 μL stop solution (cold in 4 °C, including 100 ng/mL tolbutamide and 100 ng/mL labetalol) was added to terminate the reaction. After oscillating for 10 min, the plates were centrifuged (4000 rpm) at room temperature for 20 min, and the supernatants were used for analyzation.

## 4. Conclusions 

A series of novel *N*,*N*-substituted amine derivatives were designed by utilizing bioisosterism. New compounds were synthesized and evaluated for their inhibitory activity against CETP by a BODIPY-CE fluorescence assay. Compound **17** was identified as a promising CETP inhibitor with good inhibitory activity (IC_50_ = 0.38 ± 0.08 μM), and compound **17** demonstrated weak human/rat liver microsomes stability and low CYP inhibition.

## Figures and Tables

**Table 1:**
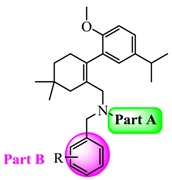
Structures and activities of compounds **12**–**22**, **32**–**37**, **40**–**44**.

NO.	Part A	R (Part B)	IC_50_ (µM)	NO.	Part A	R (Part B)	IC_50_ (µM)
**12**	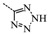	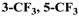	2.84 ± 0.04	**33**	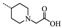	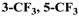	>50 ^b^
**13**	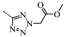	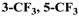	>50 ^b^	**34**	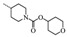	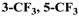	>50 ^b^
**14**	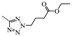	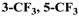	>50 ^b^	**35**	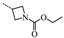	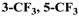	>50 ^b^
**15**	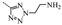	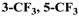	1.92 ± 0.10	**36**	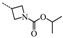	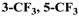	>50 ^b^
**16**	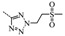	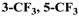	1.20 ± 0.01	**37**	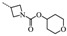	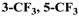	>50 ^b^
**17**	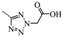	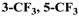	0.38 ± 0.08	**40**	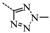	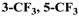	0.81 ± 0.03
**18**	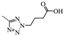	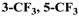	1.60 ± 0.02	**41**	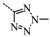	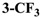	10.05 ± 0.06
**19**	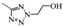	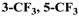	0.73 ± 0.09	**42**	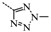	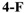	>50 ^b^
**20**	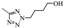	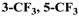	5.76 ± 0.03	**43**	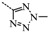	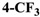	>50 ^b^
**21**	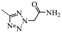	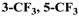	20.06 ± 0.12	**44**	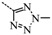	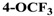	>50 ^b^
**22**	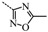	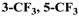	0.72 ± 0.06				
**32**	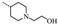	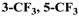	>50 ^b^	**Anacetrapib** ^a^			0.04 ± 0.01

^a^ Used as a positive control. ^b^ Considered with no CETP inhibition activity.

**Table 2:** In vitro DMPK profile.

Compound	HLM Stability ^a^ (μL/min/mg)	RLM Stability ^b^ (μL/min/mg)	CYPs Direct Inhibition Mean (10 μM)				
			3A4	2D6	2C9	1A2	2C19
**17**	119.0	146.1	17.7%	No inhibition	14.3%	No inhibition	3.1%

^a^ Human liver microsomal intrinsic clearance. ^b^ Rat liver microsomal intrinsic clearance.

## Supplementary Materials

Supplementary File 1
