# Supplementary material for: Design, Synthesis and Biological Evaluation of N,N-Substituted Amine Derivatives as Cholesteryl Ester Transfer Protein Inhibitors
Source: Molecules. 2017 Oct 3;22(10):1658. doi: 10.3390/molecules22101658 (PMC6151529; doi:10.3390/molecules22101658)
Supplement: Supplementary File 1 [file molecules-22-01658-s001.pdf]

Design, synthesis and biological evaluation of *N,N*-substituted amine derivatives as cholesteryl ester transfer protein inhibitors

Xinran Wang [1], Lijuan Hao [1], Xuanqi Xu [2], Wei Li [1], Chunchi Liu [1], Dongmei Zhao [1,*] and Maosheng Cheng [1]

[1] Key Laboratory of Structure-Based Drug Design & Discovery of Ministry of Education, Shenyang Pharmaceutical University, shenyang 110016, China; medchemzhao@163.com

[2] Department of Chemistry, University of Wisconsin- Madison, Madison, WI, USA; xxu53@wisc.edu

* Correspondence: medchemzhao@163.com; Tel.: +86-24-43520219

Content: ^{1}H NMR, ^{13}C NMR, HRMS for all target compounds reported in this study.

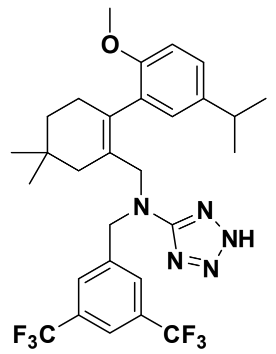
O
N
N
NH
N
N
F3C
CF3

Compound 12

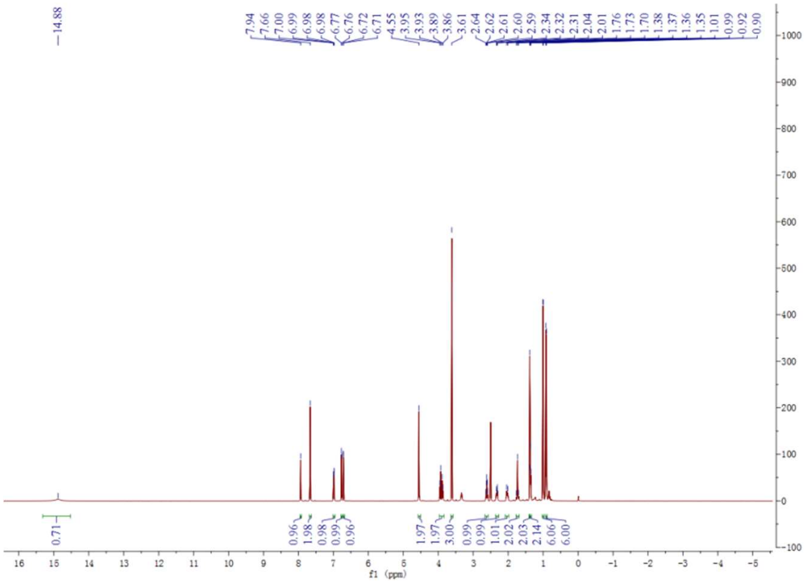
14.88
7.94
7.66
7.00
6.99
6.98
6.98
6.77
6.76
6.72
6.71
4.55
3.95
3.93
3.89
3.86
3.61
2.64
2.62
2.61
2.60
2.59
2.34
2.32
2.31
2.04
2.01
1.76
1.73
1.70
1.38
1.37
1.36
1.35
1.01
0.99
0.92
0.90
0.71
0.96
1.98
0.98
0.99
0.96
1.97
1.97
3.00
0.99
0.99
1.01
2.02
2.03
2.14
6.06
6.00
f1 (ppm)

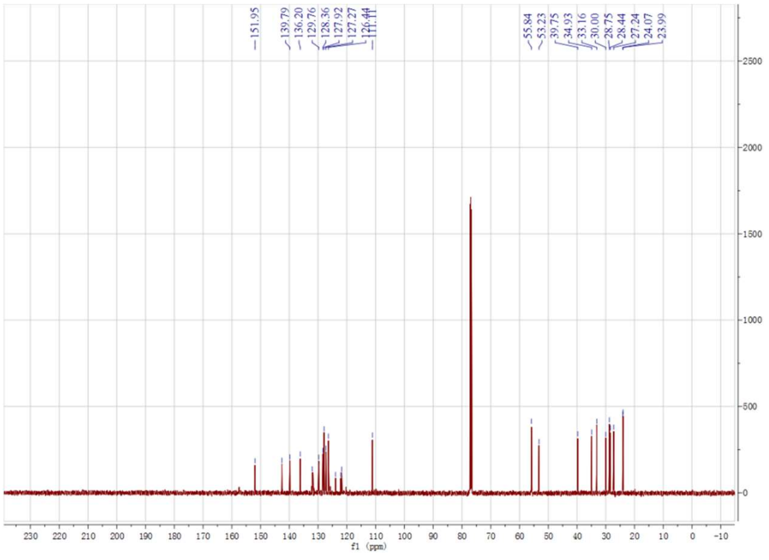
151.95
139.79
136.20
129.76
128.36
127.92
127.27
55.84
53.23
39.75
34.93
33.16
30.00
28.75
28.44
27.24
24.07
23.99
f1 (ppm)

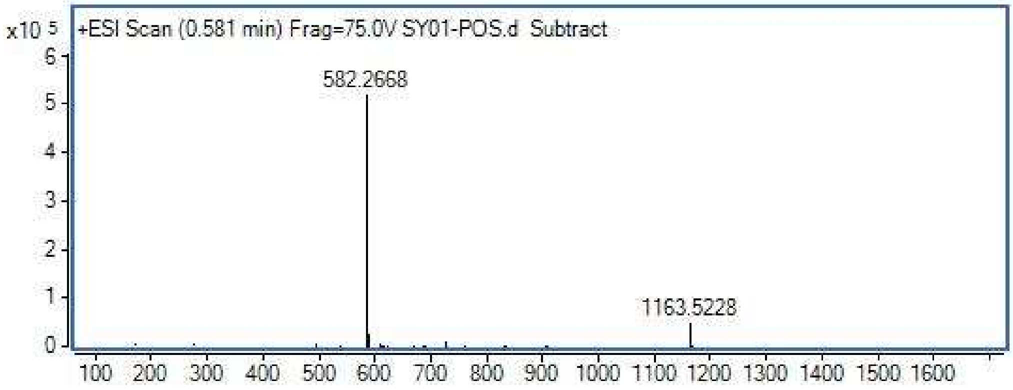
x10 5
+ESI Scan (0.581 min) Frag=75.0V SY01-POS.d Subtract
582.2668
1163.5228
0
1
2
3
4
5
6
100 200 300 400 500 600 700 800 900 1000 1100 1200 1300 1400 1500 1600

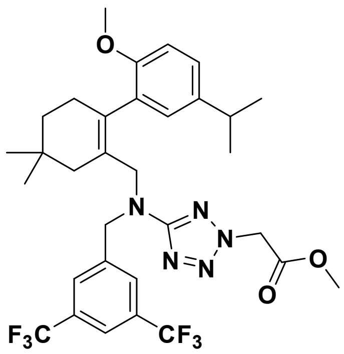

Compound 13

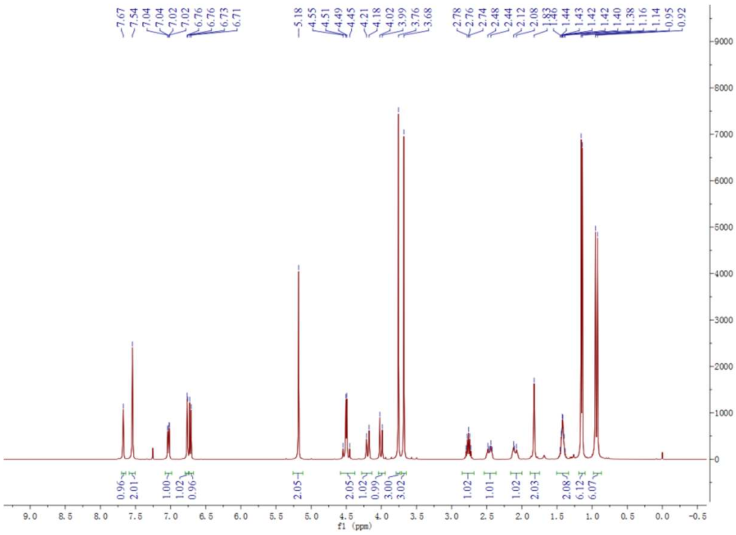

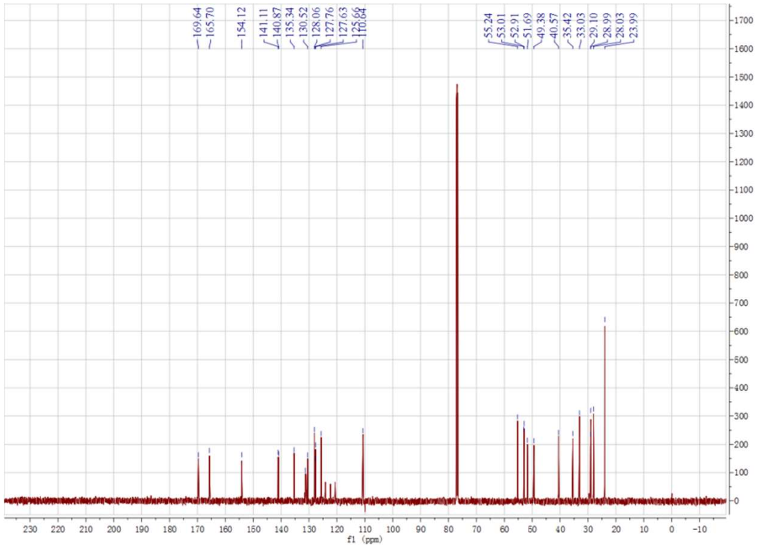

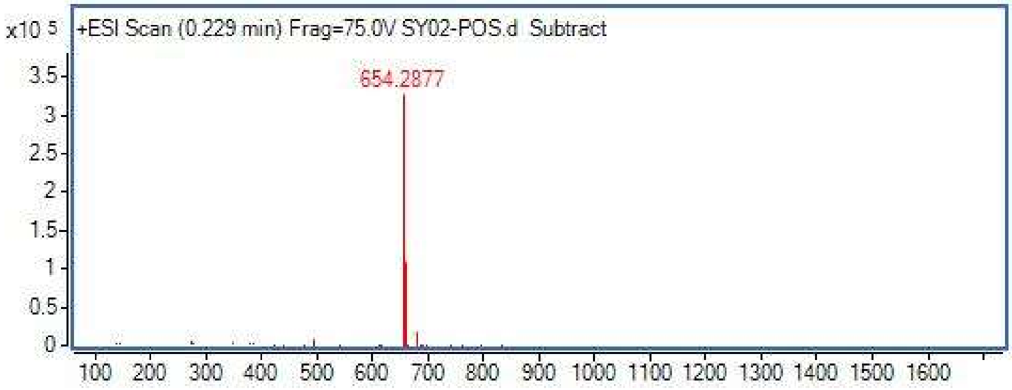

x10 5
+ESI Scan (0.229 min) Frag=75.0V SY02-POS.d Subtract
654.2877
3.5
3
2.5
2
1.5
1
0.5
0
100 200 300 400 500 600 700 800 900 1000 1100 1200 1300 1400 1500 1600

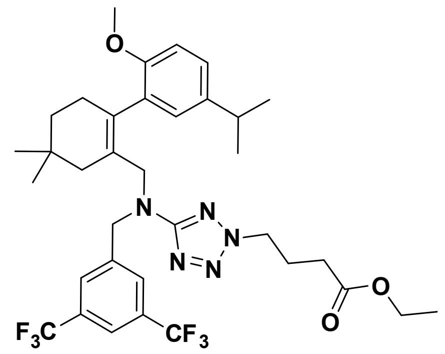

Compound 14

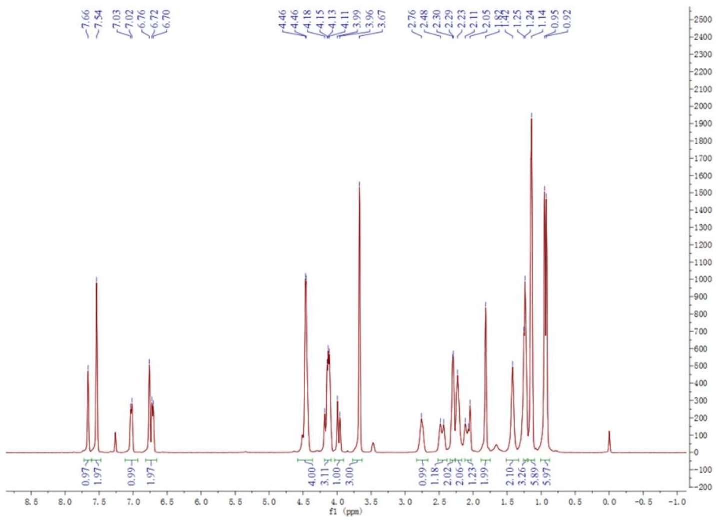

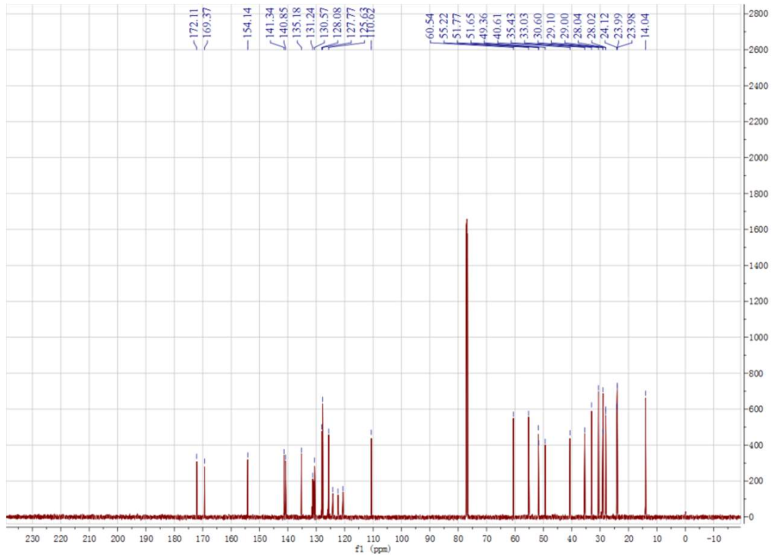

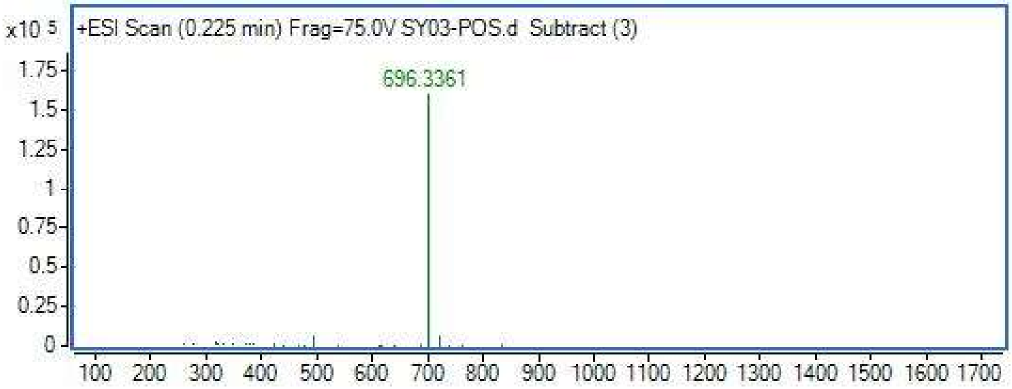
x10 5
+ESI Scan (0.225 min) Frag=75.0V SY03-POS.d Subtract (3)
696.3361
1.75
1.5
1.25
1
0.75
0.5
0.25
0
100 200 300 400 500 600 700 800 900 1000 1100 1200 1300 1400 1500 1600 1700

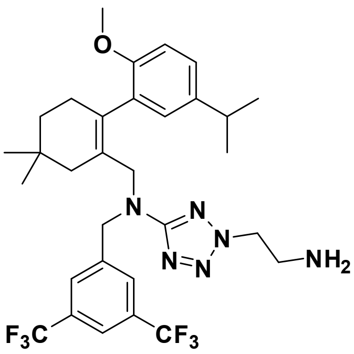

Compound 15

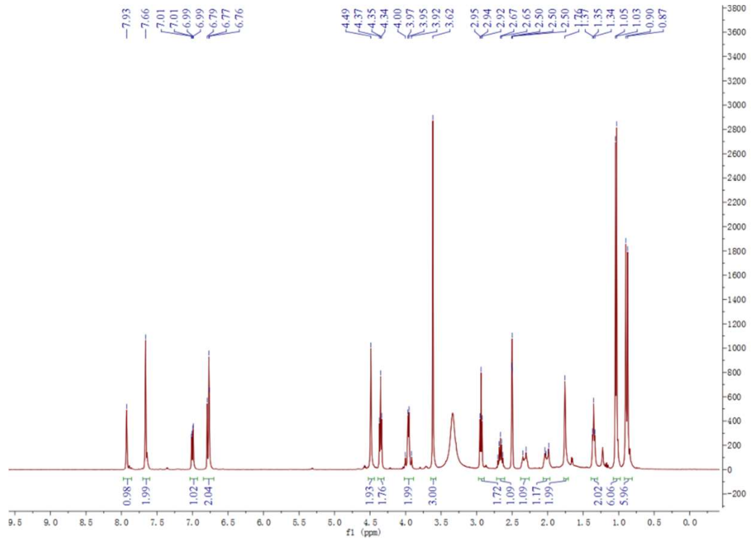
7.93
7.66
7.01
7.01
6.99
6.99
6.79
6.77
6.76
4.49
4.37
4.35
4.34
4.00
3.97
3.95
3.92
3.62
2.95
2.94
2.92
2.67
2.65
2.50
2.50
2.50
1.35
1.34
1.05
1.03
0.90
0.87
0.98
1.99
1.02
2.04
1.93
1.76
1.99
3.00
1.72
1.09
1.09
1.17
1.99
2.02
6.06
5.96
f1 (ppm)

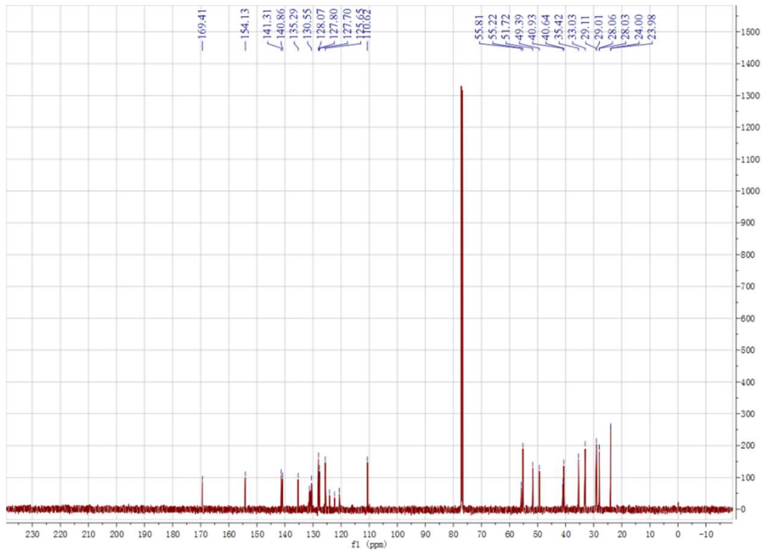
169.41
154.13
141.31
140.86
135.29
130.55
128.07
127.80
127.70
125.65
110.62
55.81
55.22
51.72
49.39
40.93
40.64
35.42
33.03
29.11
29.01
28.06
28.03
24.00
23.98
f1 (ppm)

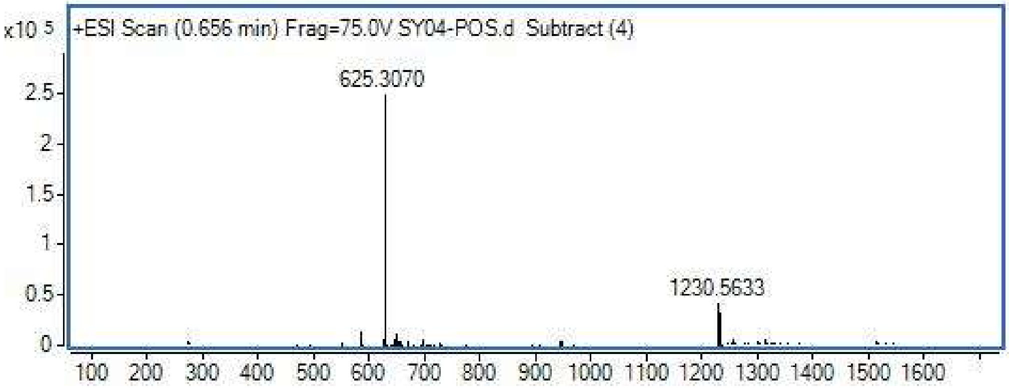
x10 5
+ESI Scan (0.656 min) Frag=75.0V SY04-POS.d Subtract (4)
625.3070
1230.5633
2.5
2
1.5
1
0.5
0
100 200 300 400 500 600 700 800 900 1000 1100 1200 1300 1400 1500 1600

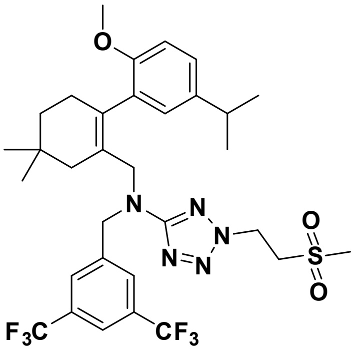

Compound 16

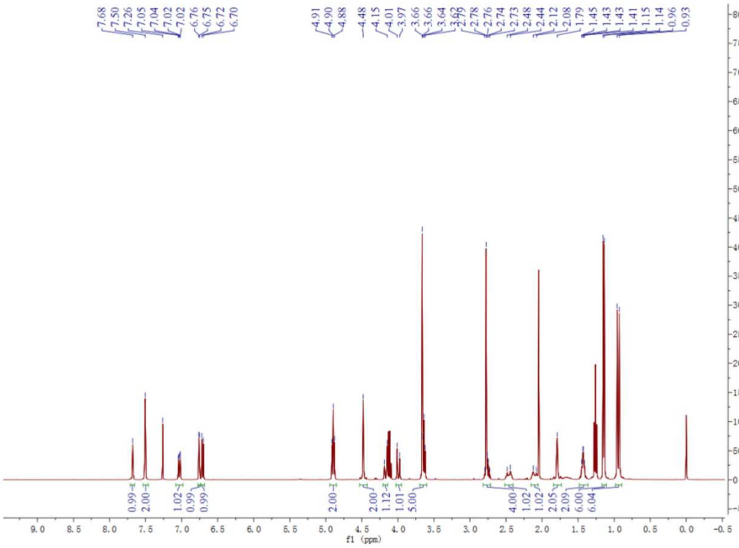
7.68 7.50 7.26 7.05 7.04 7.02 7.02 6.76 6.75 6.72 6.70
4.91 4.90 4.88 4.48 4.15 4.01 3.97 3.66 3.66 3.64 3.62 2.79 2.78 2.76 2.74 2.73 2.48 2.44 2.12 2.08 1.79 1.45 1.43 1.43 1.41 1.15 1.14 0.96 0.93
0.99 2.00 1.02 0.99 0.99 2.00 2.00 1.12 1.01 5.00 4.00 1.02 1.02 2.05 2.09 6.00 6.04
f1 (ppm)

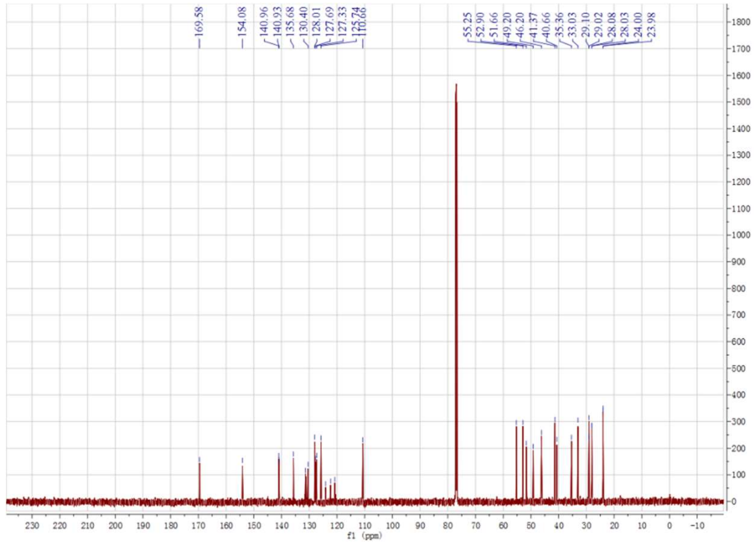
169.58 154.08 140.96 140.93 135.68 130.40 128.01 127.69 127.33 125.74 110.66
55.25 52.90 51.66 49.20 46.20 41.37 40.66 35.36 33.03 29.10 29.02 28.08 28.03 24.00 23.98
f1 (ppm)

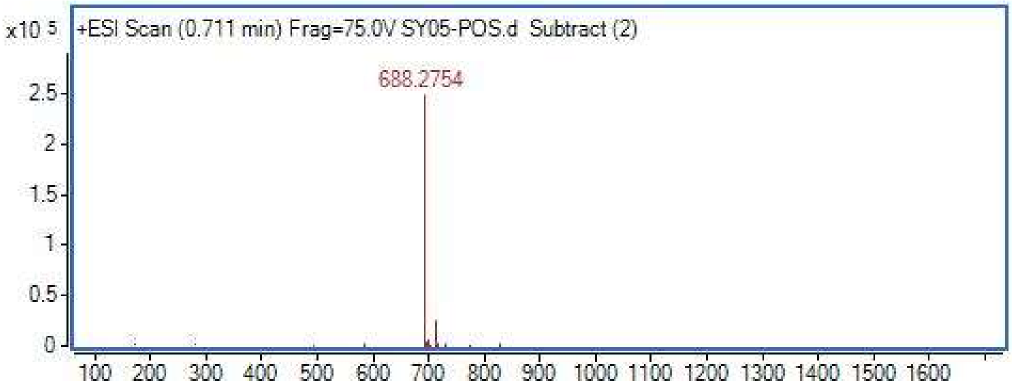
x10 5
+ESI Scan (0.711 min) Frag=75.0V SY05-POS.d Subtract (2)
688.2754
2.5
2
1.5
1
0.5
0
100 200 300 400 500 600 700 800 900 1000 1100 1200 1300 1400 1500 1600

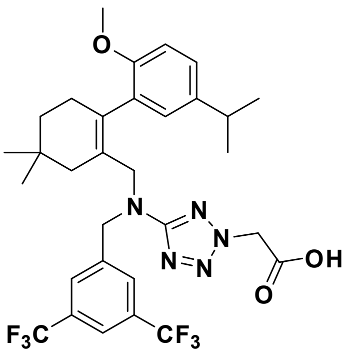
O
N
N
N
N
N
OH
O
F3C
CF3
Compound 17

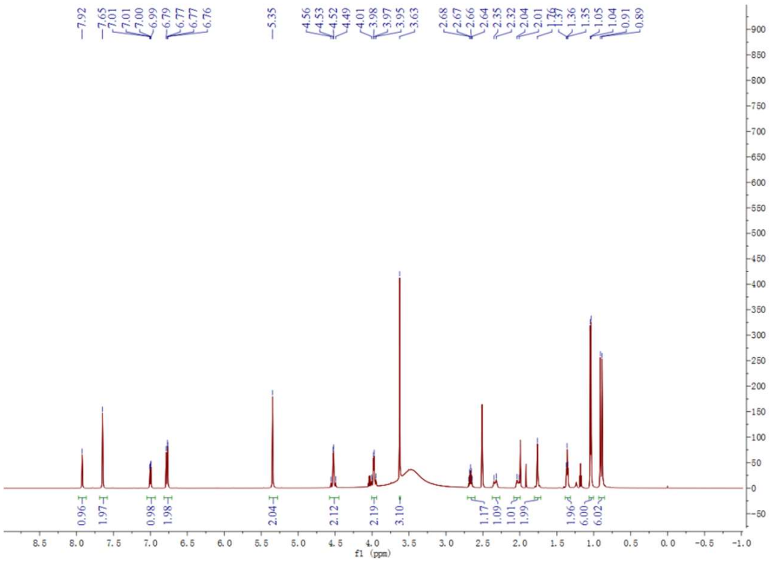
7.92
7.65
7.01
7.01
7.00
6.99
6.79
6.77
6.77
6.76
5.35
4.56
4.53
4.52
4.49
4.01
3.98
3.97
3.95
3.63
2.68
2.67
2.66
2.64
2.35
2.32
2.04
2.01
1.76
1.37
1.36
1.35
1.05
1.04
0.91
0.89
0.96
1.97
0.98
1.98
2.04
2.12
2.19
3.10
1.17
1.09
1.01
1.99
1.96
6.00
6.02
f1 (ppm)

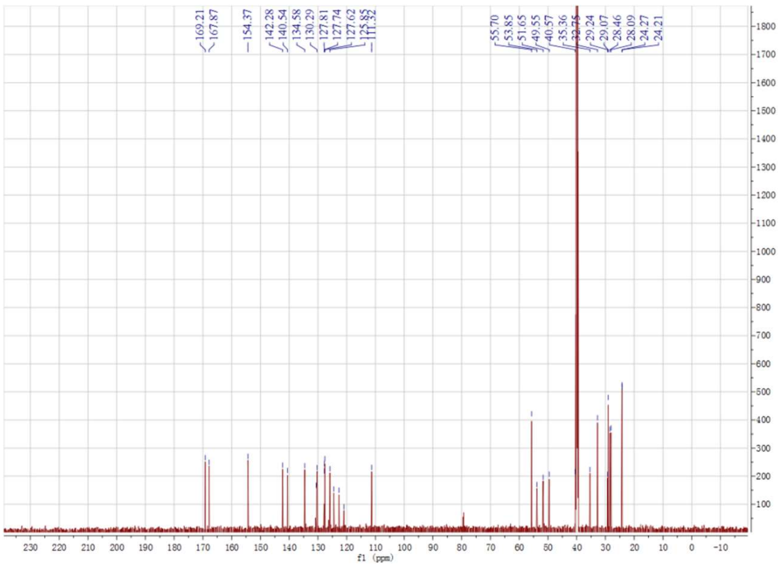
169.21
167.87
154.37
142.28
140.54
134.58
130.29
127.81
127.74
127.62
125.85
111.32
55.70
53.85
51.65
49.55
40.57
35.36
29.24
29.07
28.46
28.09
24.27
24.21
f1 (ppm)

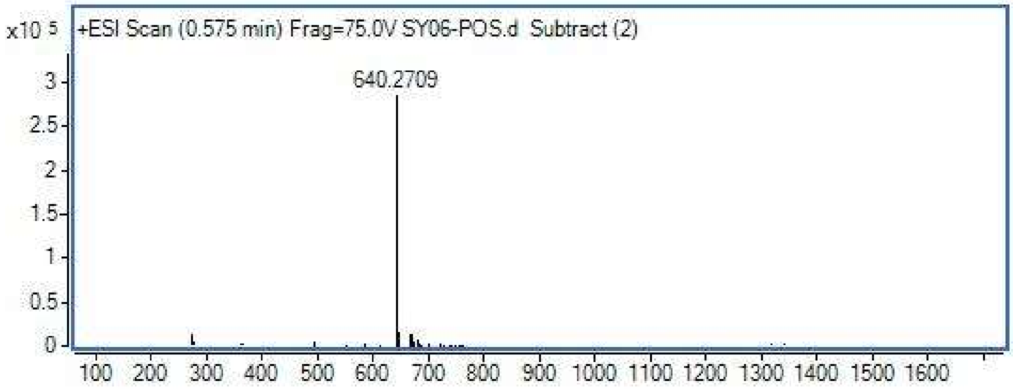
x10 5
+ESI Scan (0.575 min) Frag=75.0V SY06-POS.d Subtract (2)
640.2709
3
2.5
2
1.5
1
0.5
0
100 200 300 400 500 600 700 800 900 1000 1100 1200 1300 1400 1500 1600

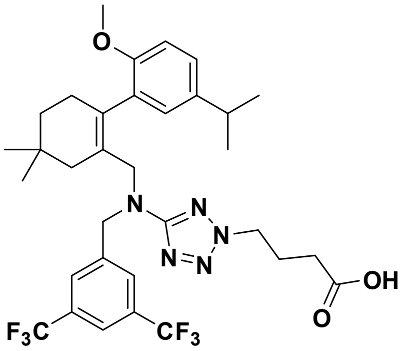

Compound 18

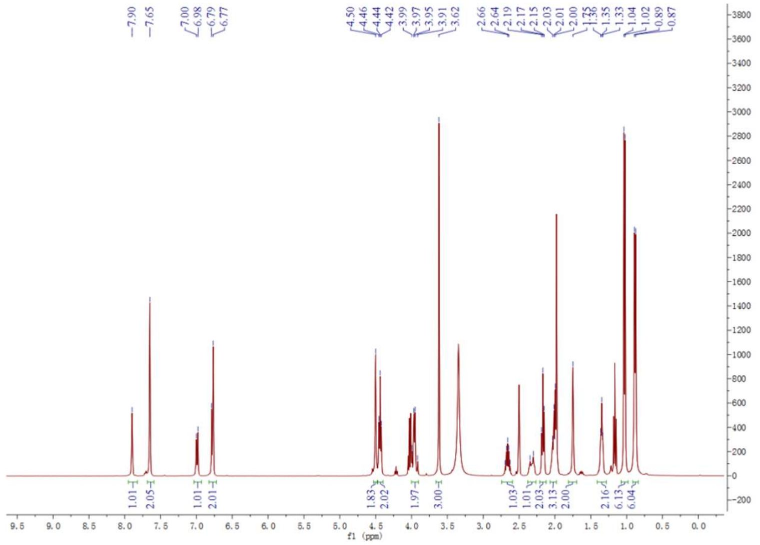

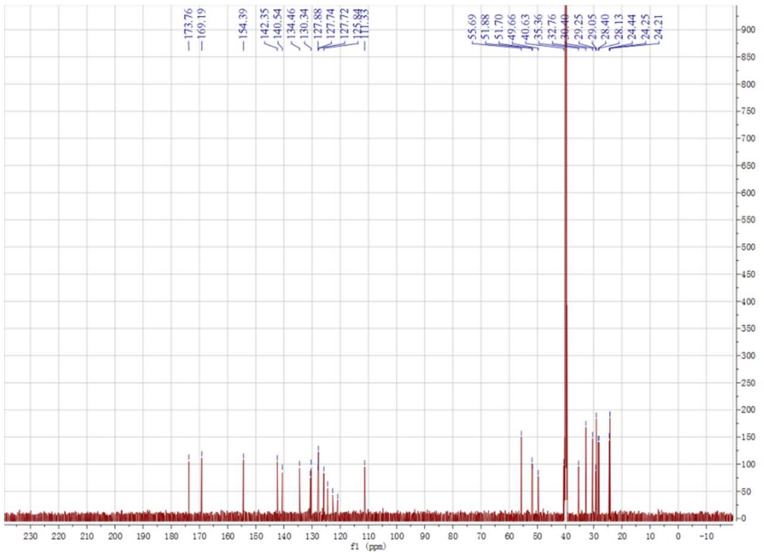

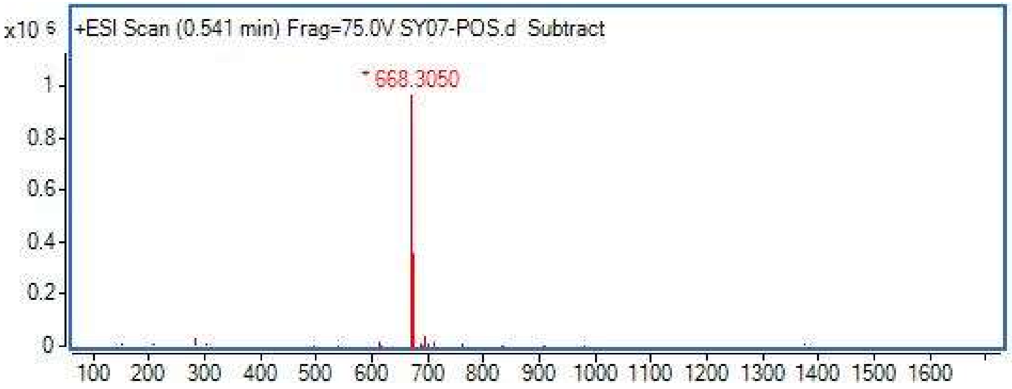
x10 6
+ESI Scan (0.541 min) Frag=75.0V SY07-POS.d Subtract
668.3050
1
0.8
0.6
0.4
0.2
0
100 200 300 400 500 600 700 800 900 1000 1100 1200 1300 1400 1500 1600

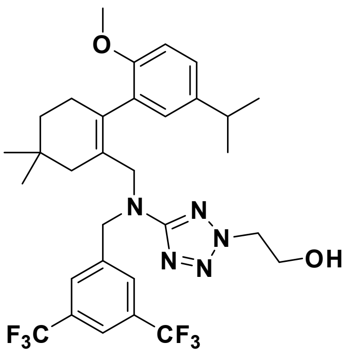

Compound 19

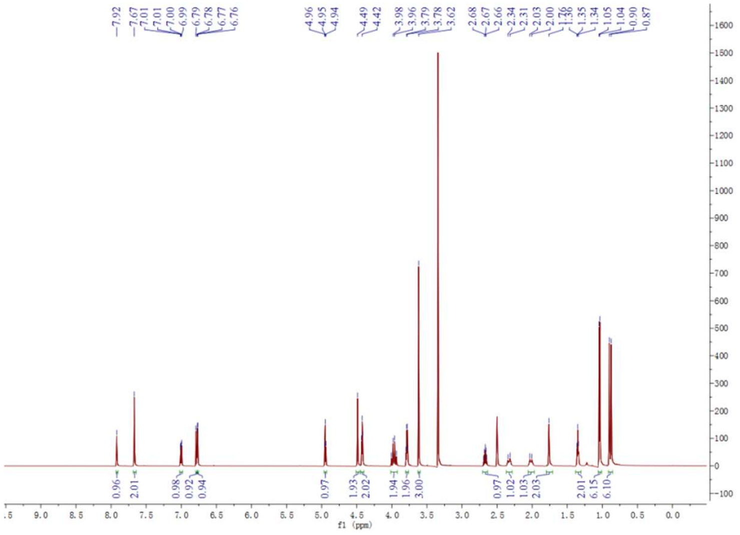

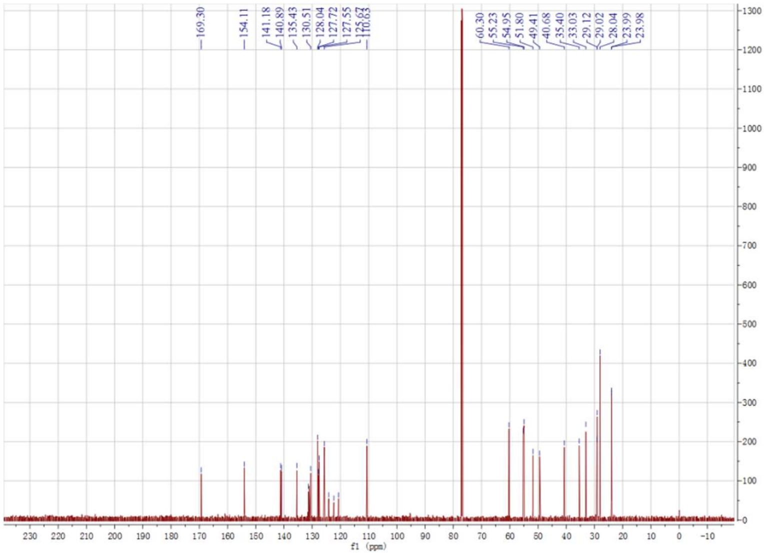

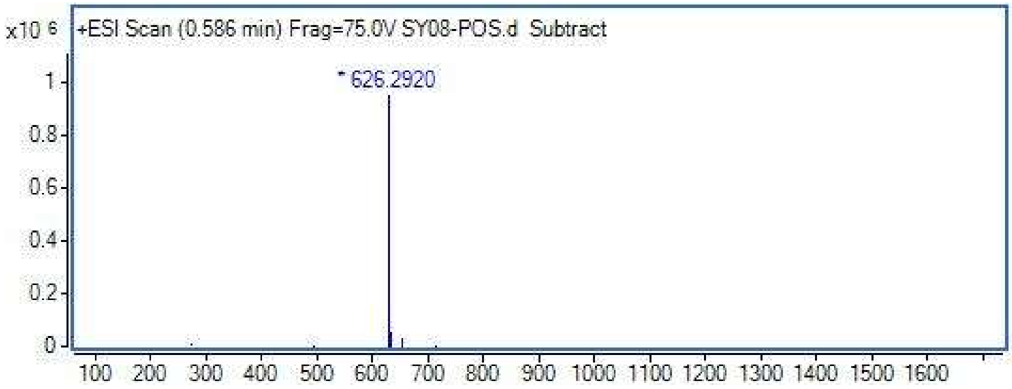
x10 6
+ESI Scan (0.586 min) Frag=75.0V SY08-POS.d Subtract
* 626.2920
1
0.8
0.6
0.4
0.2
0
100 200 300 400 500 600 700 800 900 1000 1100 1200 1300 1400 1500 1600

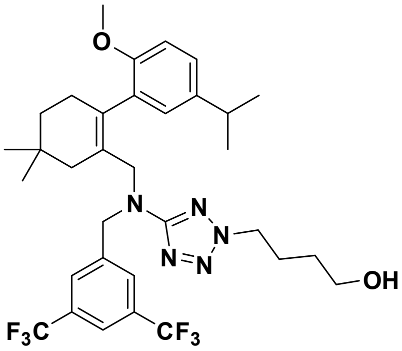
O
N
N
N
N
N
OH
F3C
CF3
Compound 20

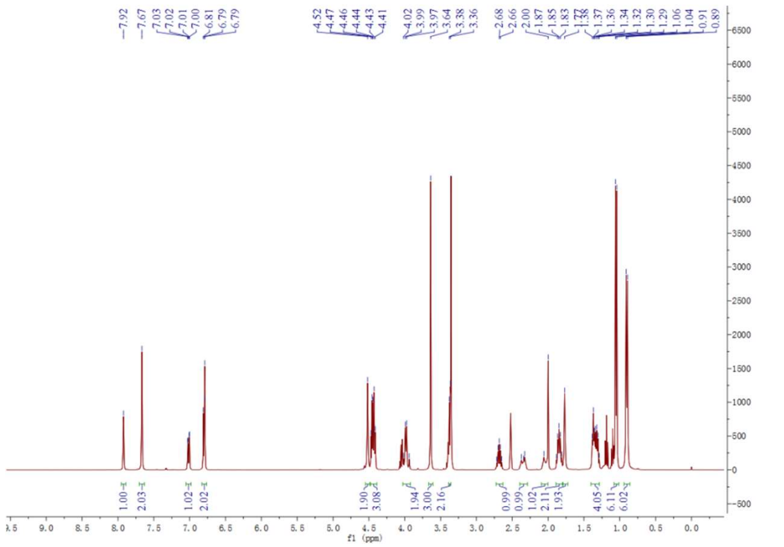
7.92
7.67
7.03
7.02
7.01
7.00
6.81
6.79
6.79
4.52
4.47
4.46
4.44
4.43
4.41
4.02
3.99
3.97
3.64
3.38
3.36
2.68
2.66
2.00
1.87
1.85
1.83
1.37
1.36
1.34
1.32
1.30
1.29
1.06
1.04
0.91
0.89
1.00
2.03
1.02
2.02
1.90
3.08
1.94
3.00
2.16
0.99
0.99
1.02
2.11
1.93
4.05
6.11
6.02
f1 (ppm)

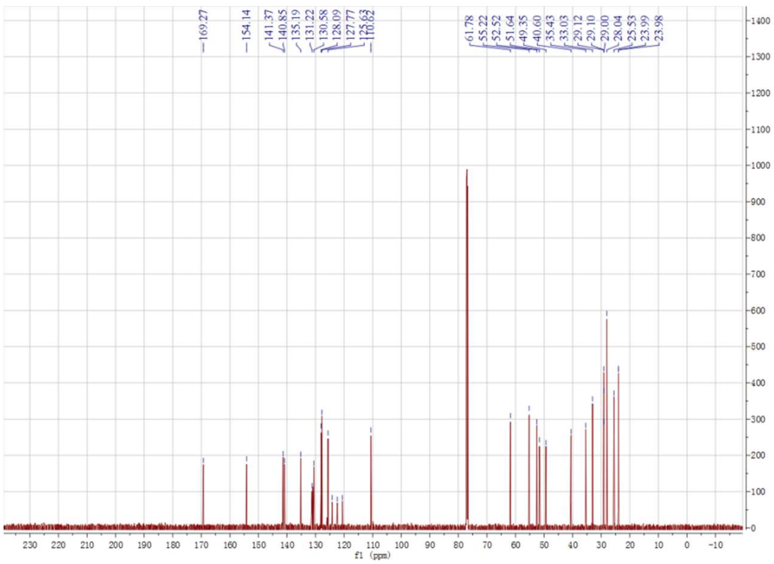
169.27
154.14
141.37
140.85
135.19
131.22
130.58
128.09
127.77
125.63
110.62
61.78
55.22
52.52
51.64
49.35
40.60
35.43
33.03
29.12
29.10
29.00
28.04
25.53
23.99
23.98
f1 (ppm)

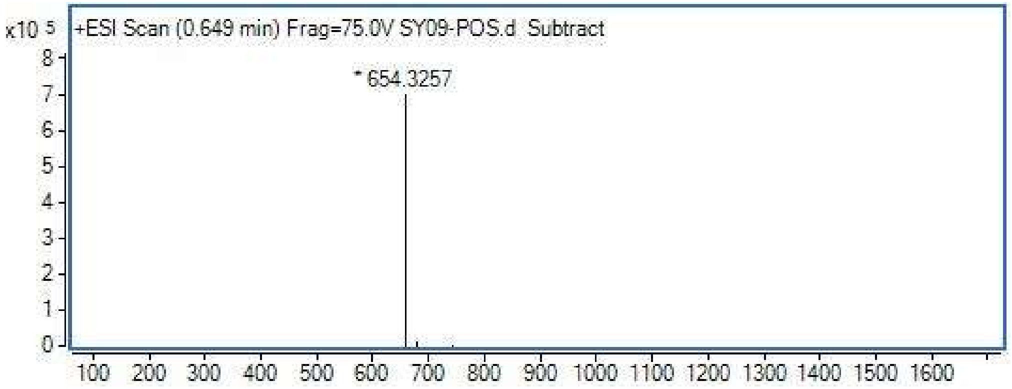
x10 5
+ESI Scan (0.649 min) Frag=75.0V SY09-POS.d Subtract
* 654.3257
8
7
6
5
4
3
2
1
0
100 200 300 400 500 600 700 800 900 1000 1100 1200 1300 1400 1500 1600

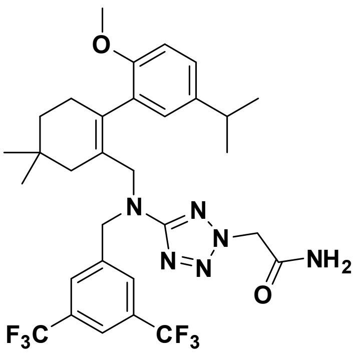

Compound 21

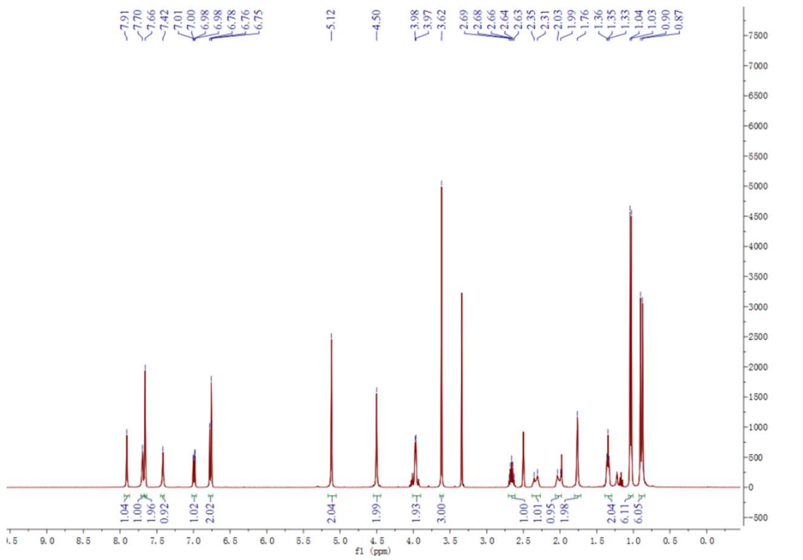

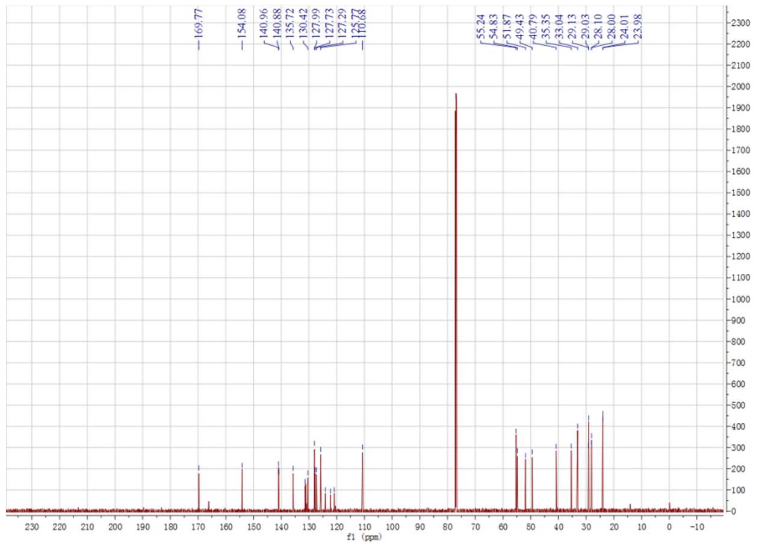

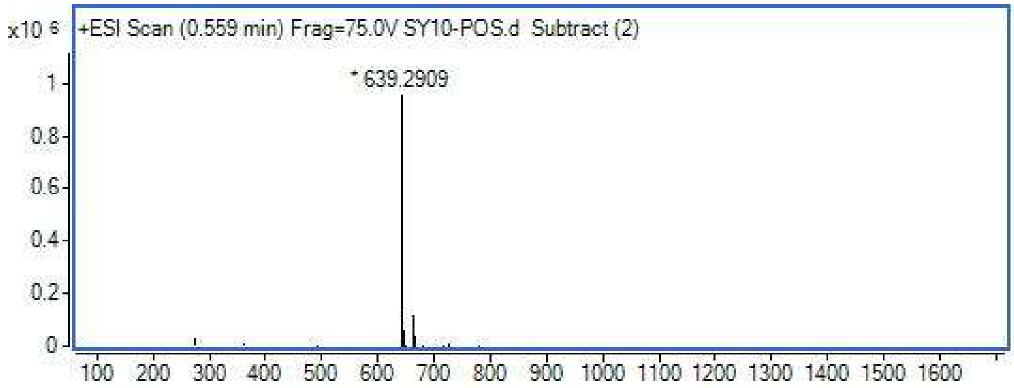
x10 6
+ESI Scan (0.559 min) Frag=75.0V SY10-POS.d Subtract (2)
* 639.2909
1
0.8
0.6
0.4
0.2
0
100 200 300 400 500 600 700 800 900 1000 1100 1200 1300 1400 1500 1600

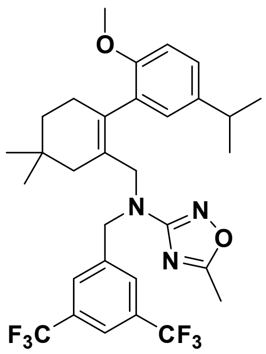

Compound 22

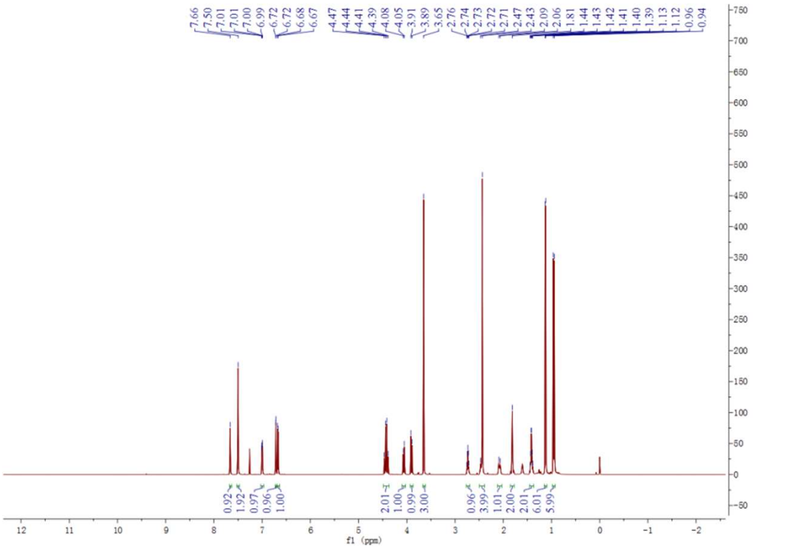

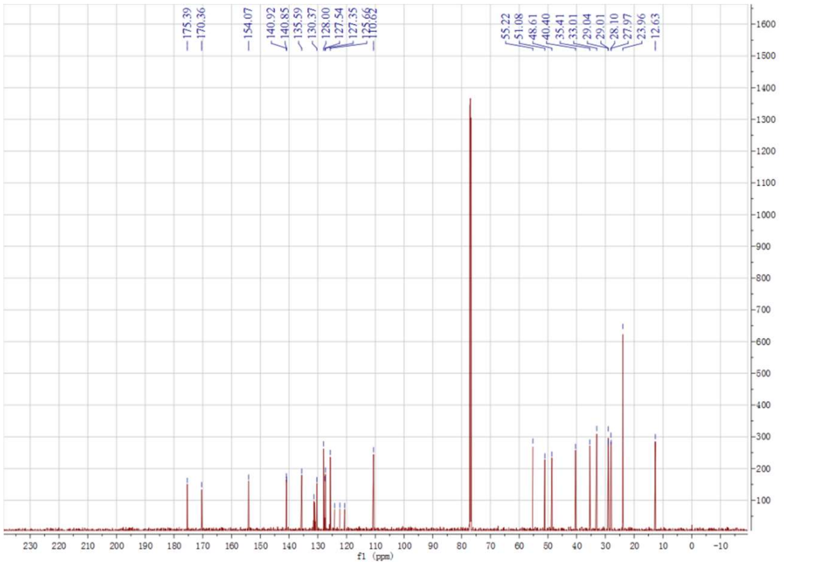

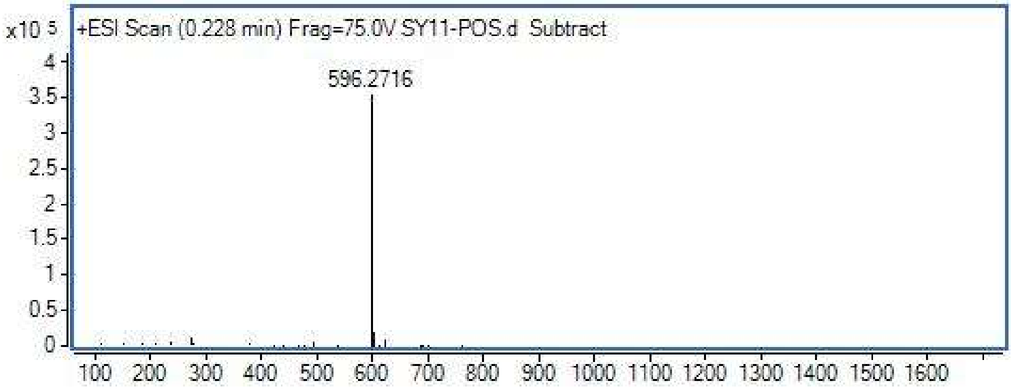
x10 5
+ESI Scan (0.228 min) Frag=75.0V SY11-POS.d Subtract
596.2716
4
3.5
3
2.5
2
1.5
1
0.5
0
100 200 300 400 500 600 700 800 900 1000 1100 1200 1300 1400 1500 1600

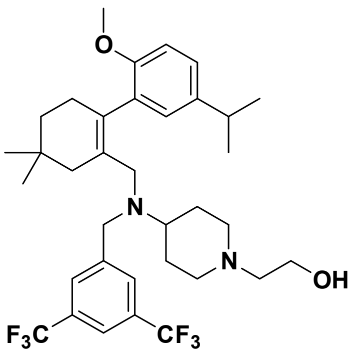

Compound 32

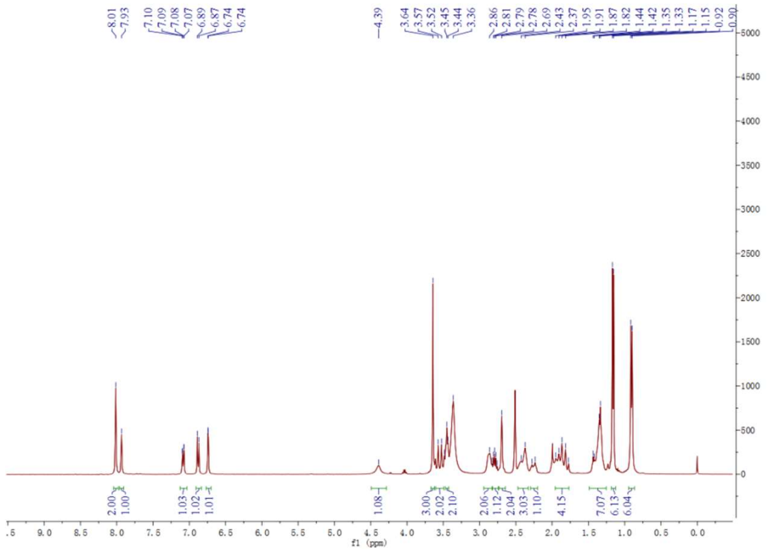

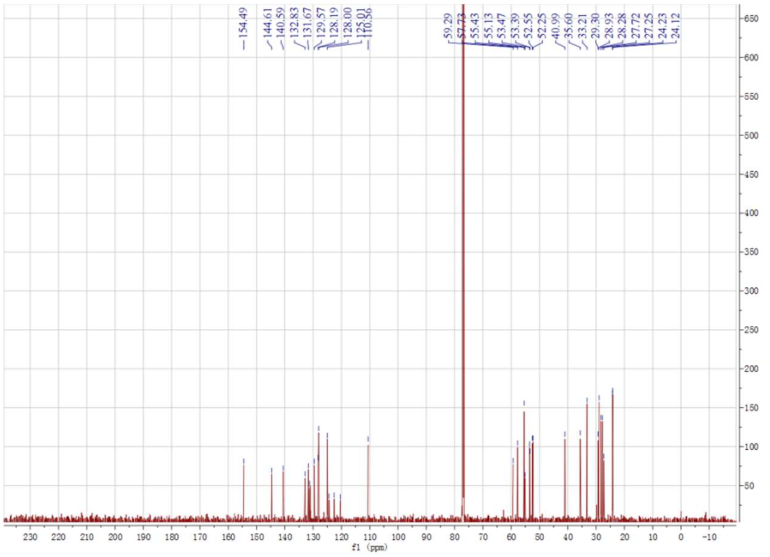

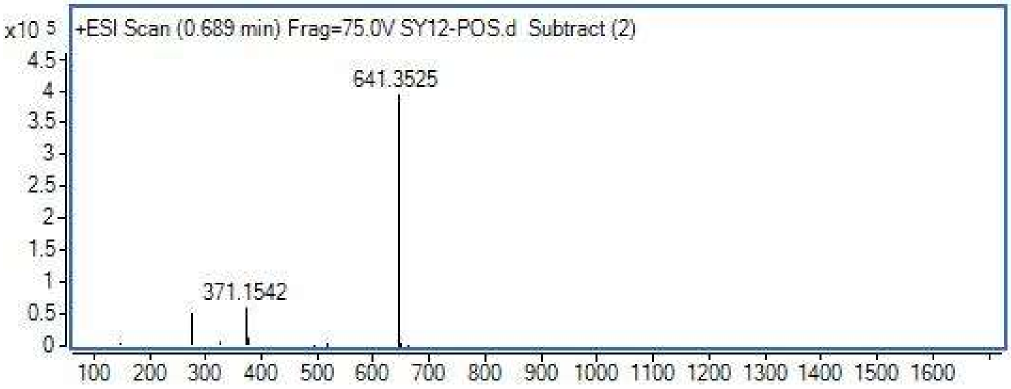

x10 5
+ESI Scan (0.689 min) Frag=75.0V SY12-POS.d Subtract (2)
641.3525
371.1542
4.5
4
3.5
3
2.5
2
1.5
1
0.5
0
100 200 300 400 500 600 700 800 900 1000 1100 1200 1300 1400 1500 1600

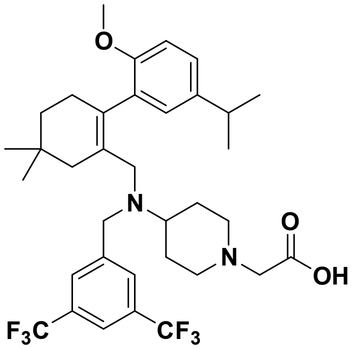

Compound 33

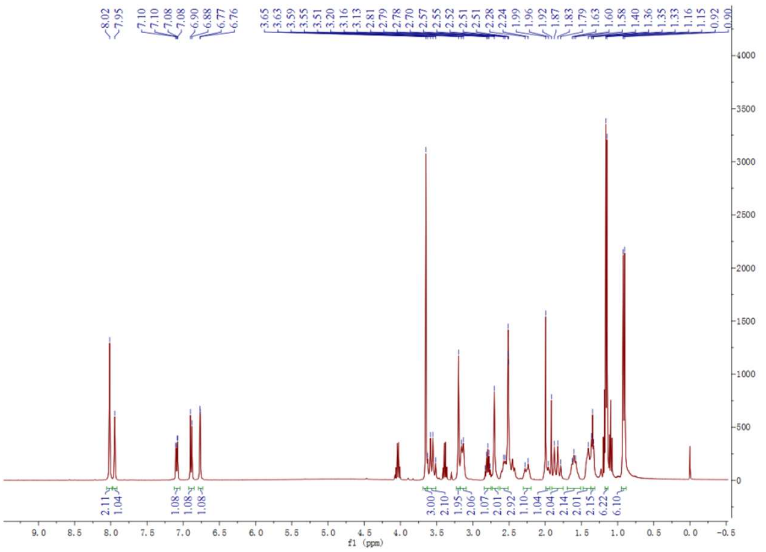

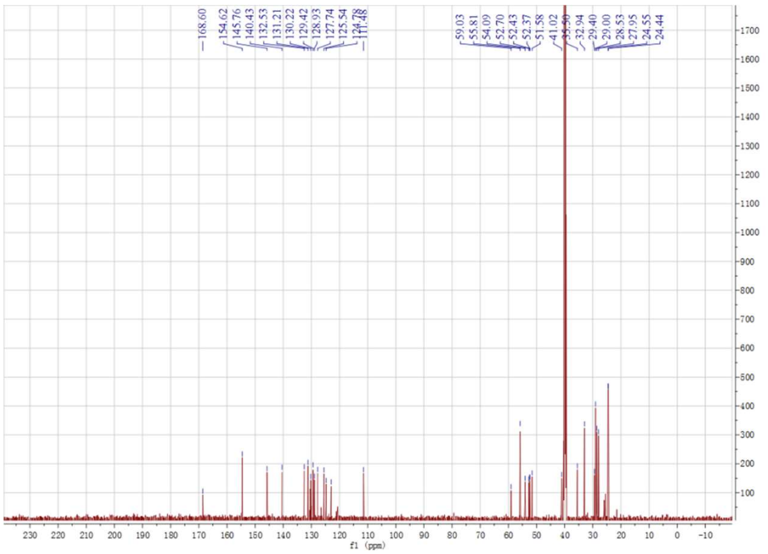

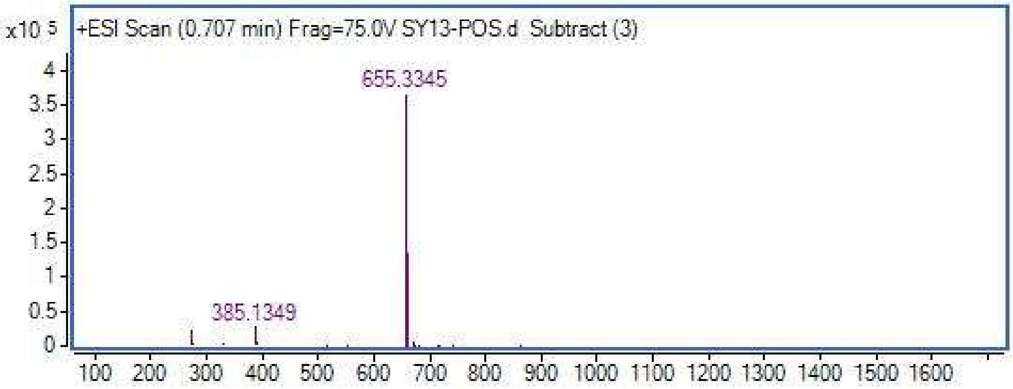
x10 5
+ESI Scan (0.707 min) Frag=75.0V SY13-POS.d Subtract (3)
655.3345
385.1349
4
3.5
3
2.5
2
1.5
1
0.5
0
100 200 300 400 500 600 700 800 900 1000 1100 1200 1300 1400 1500 1600

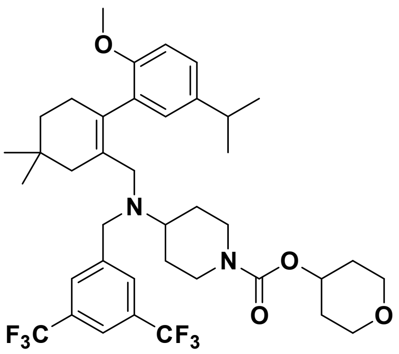

Compound 34

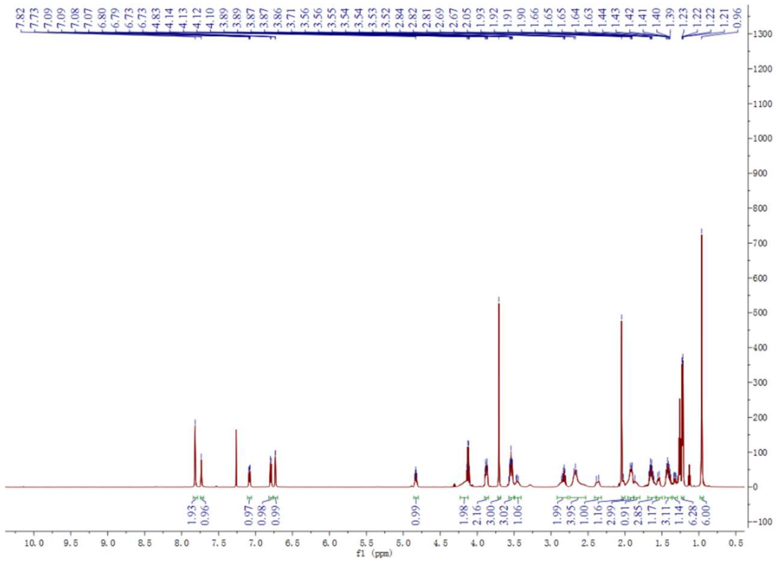

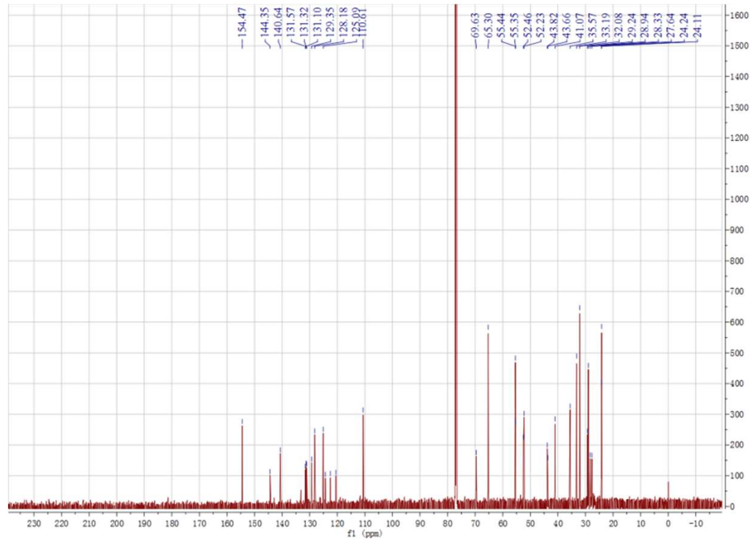

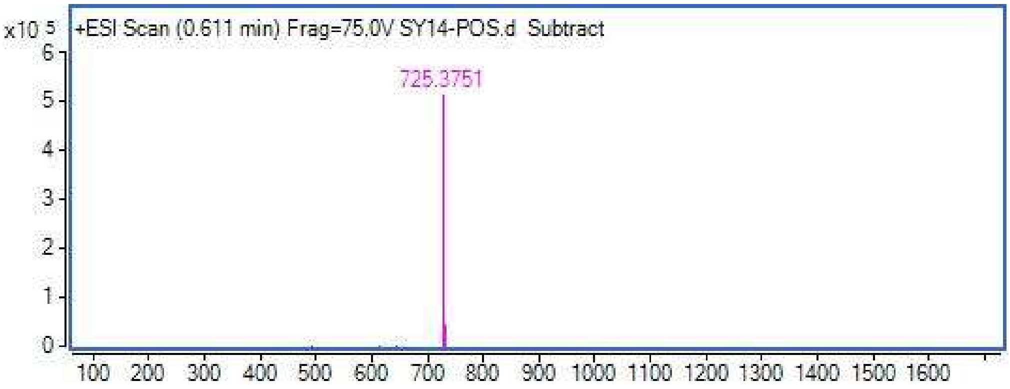
x10 5
+ESI Scan (0.611 min) Frag=75.0V SY14-POS.d Subtract
725.3751
6
5
4
3
2
1
0
100 200 300 400 500 600 700 800 900 1000 1100 1200 1300 1400 1500 1600

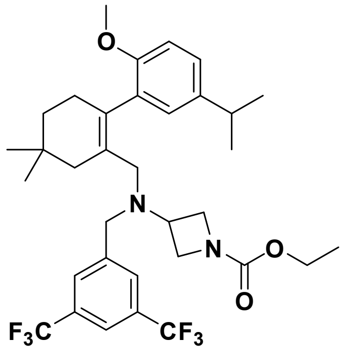

Compound 35

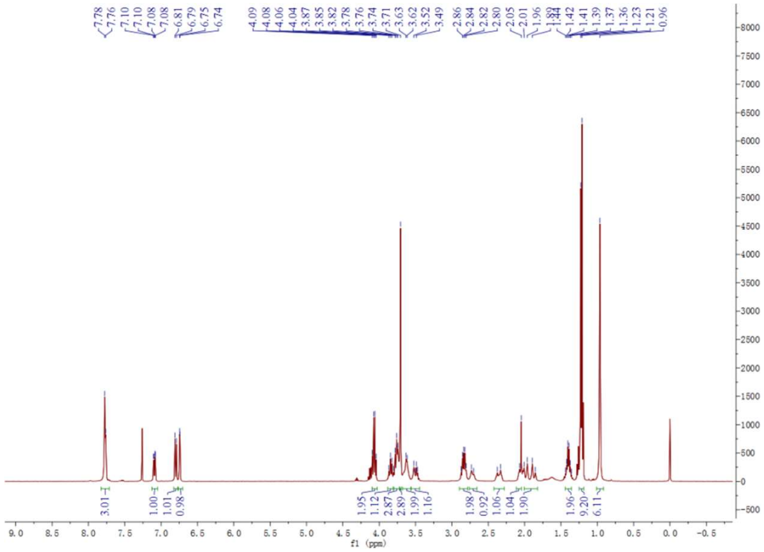

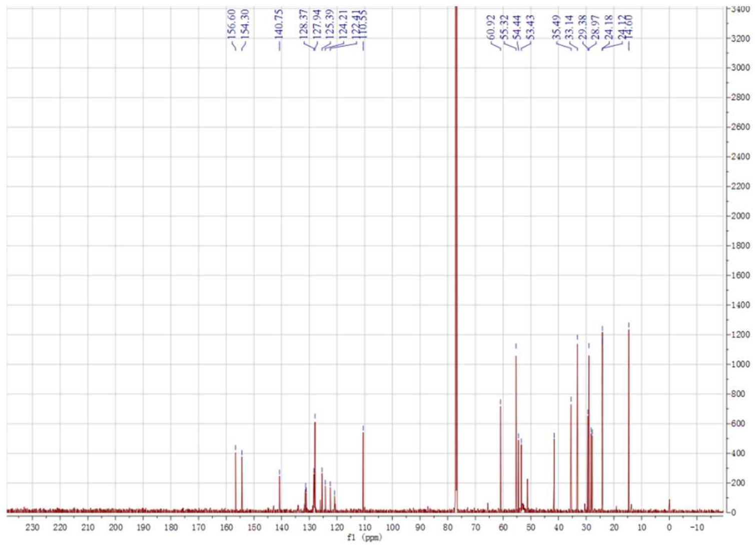

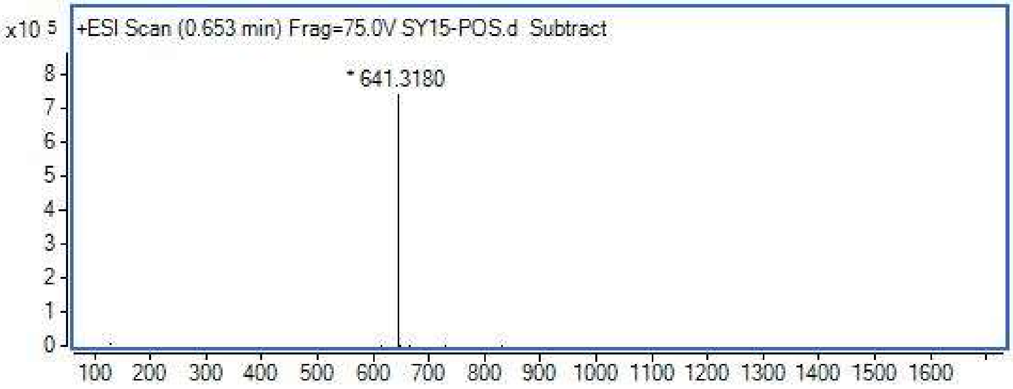
x10 5
+ESI Scan (0.653 min) Frag=75.0V SY15-POS.d Subtract
* 641.3180
8
7
6
5
4
3
2
1
0
100 200 300 400 500 600 700 800 900 1000 1100 1200 1300 1400 1500 1600

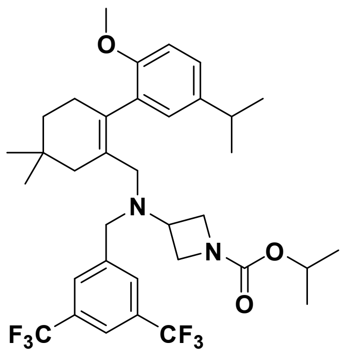

Compound 36

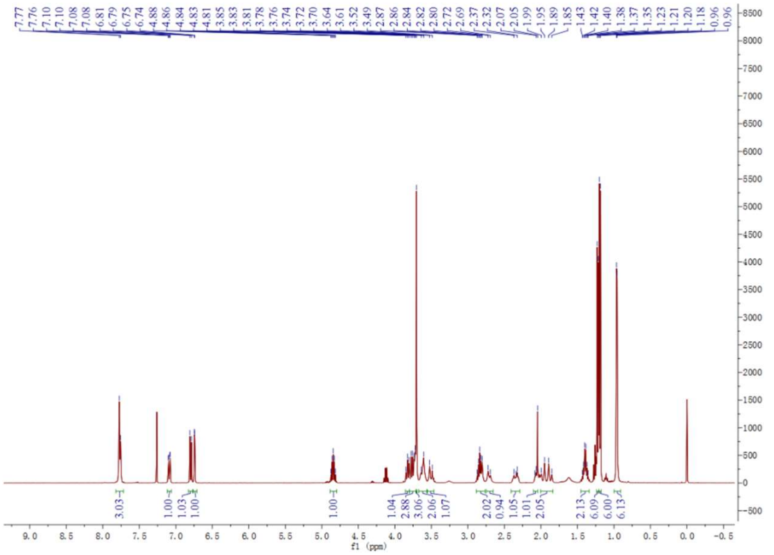

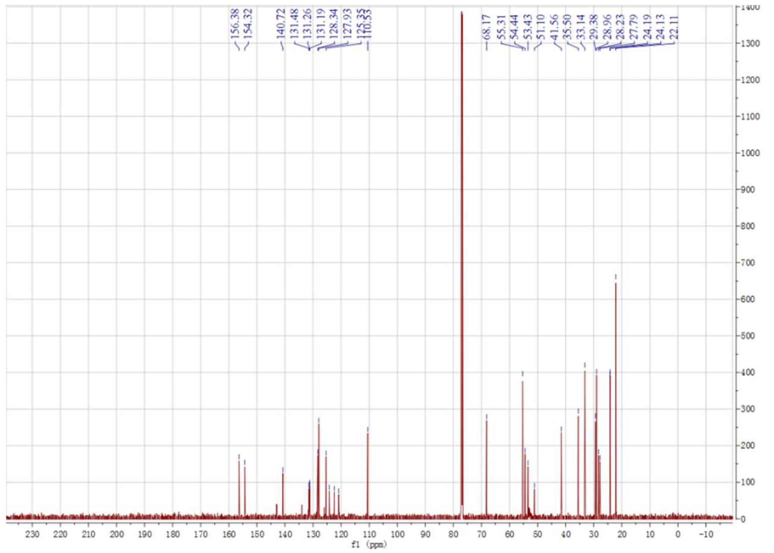

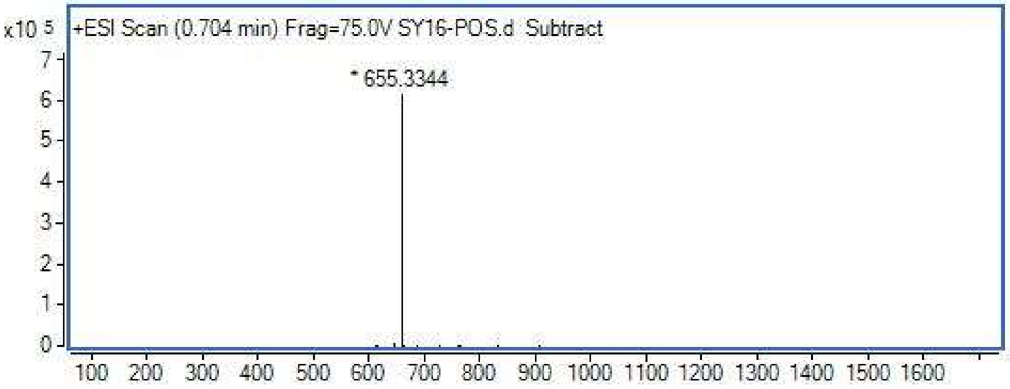

x10 5
+ESI Scan (0.704 min) Frag=75.0V SY16-POS.d Subtract
* 655.3344
7
6
5
4
3
2
1
0
100 200 300 400 500 600 700 800 900 1000 1100 1200 1300 1400 1500 1600

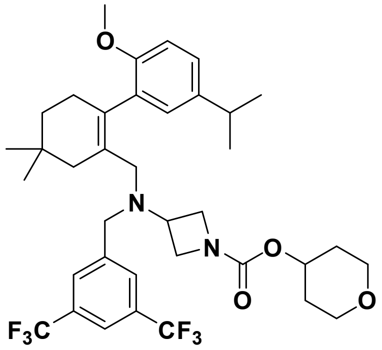

Compound 37

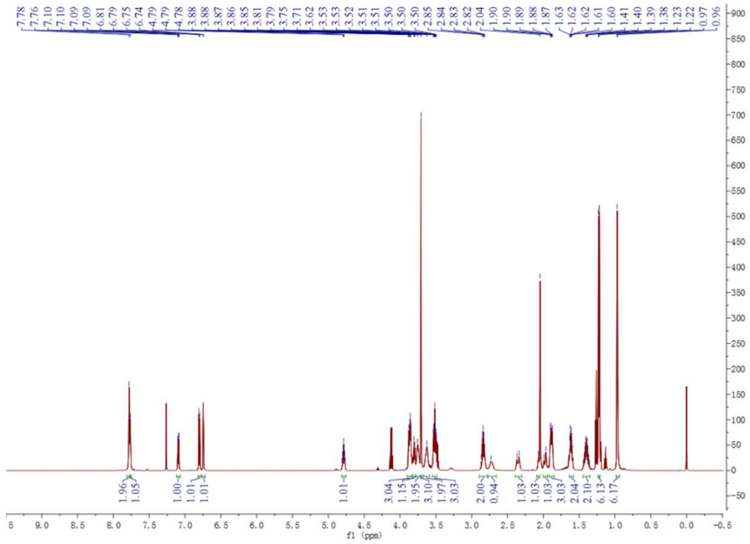

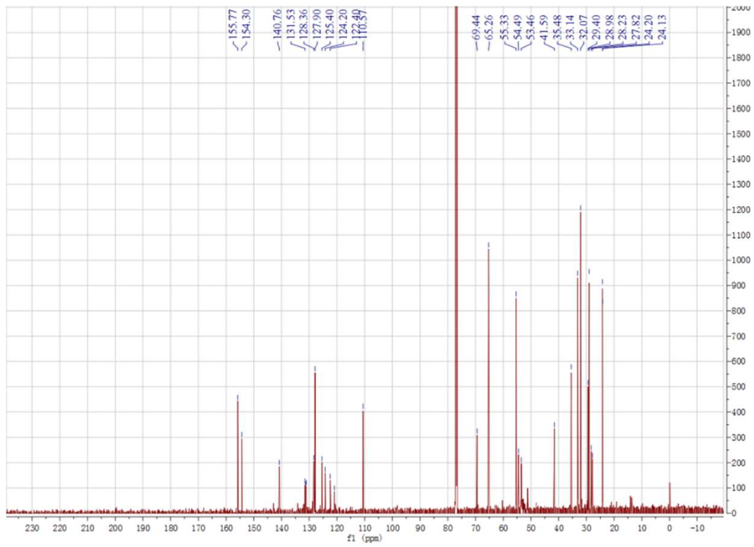

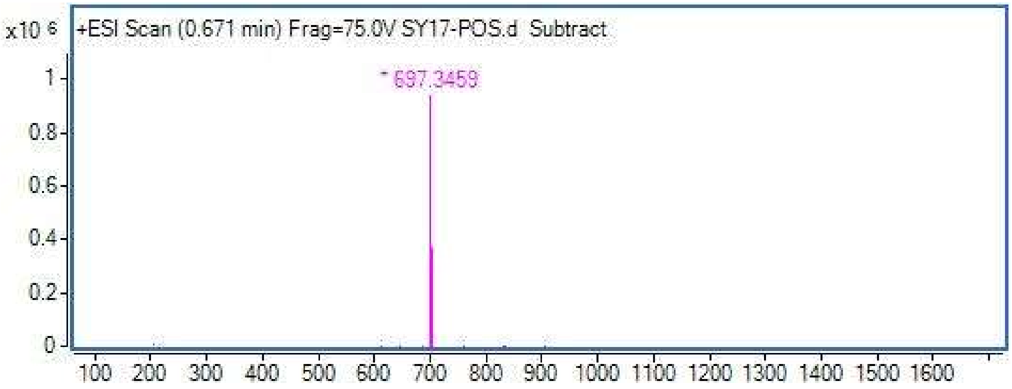
x10 6
+ESI Scan (0.671 min) Frag=75.0V SY17-POS.d Subtract
697.3459
1
0.8
0.6
0.4
0.2
0
100 200 300 400 500 600 700 800 900 1000 1100 1200 1300 1400 1500 1600

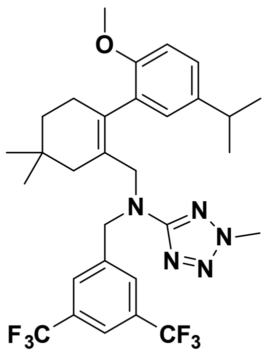

Compound 40

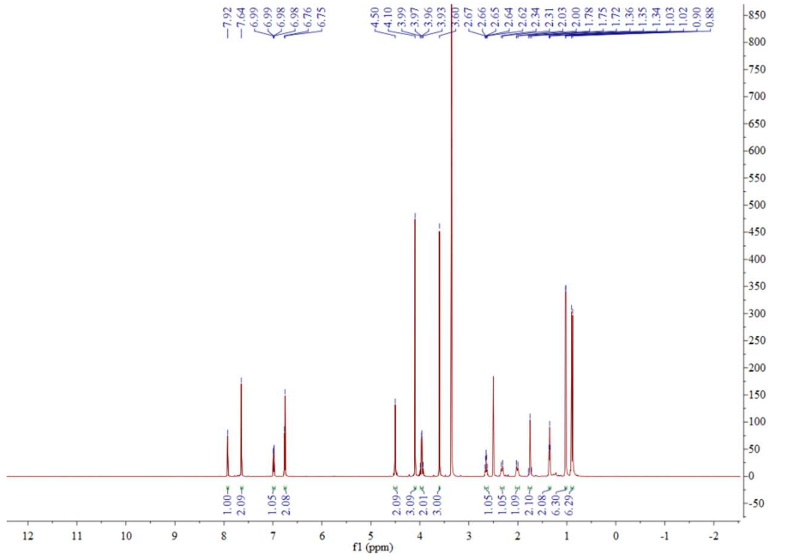

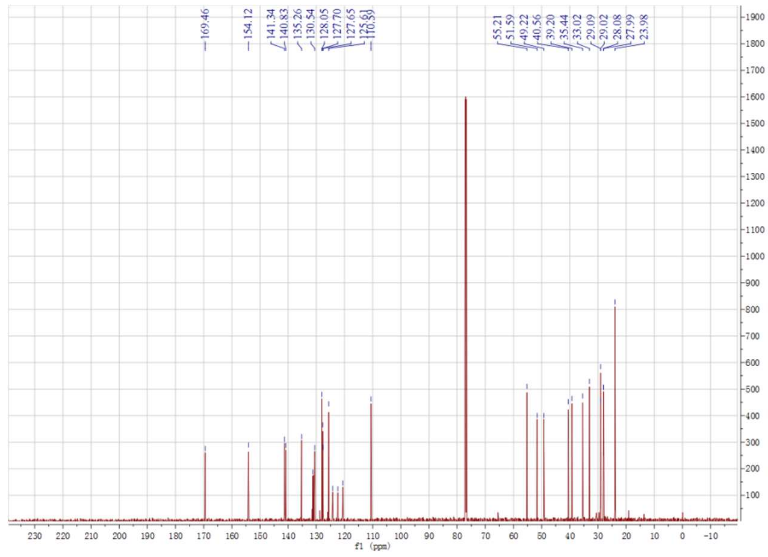

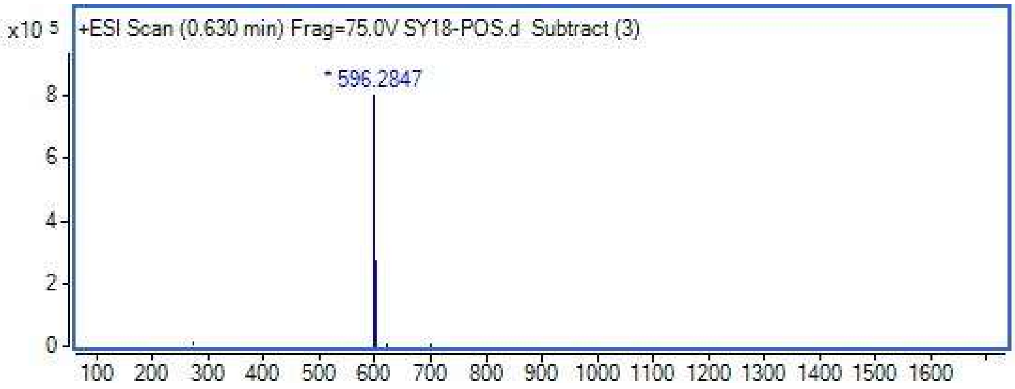
x10 5
+ESI Scan (0.630 min) Frag=75.0V SY18-POS.d Subtract (3)
* 596.2847
8
6
4
2
0
100 200 300 400 500 600 700 800 900 1000 1100 1200 1300 1400 1500 1600

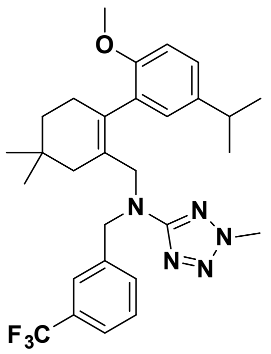

Compound 41

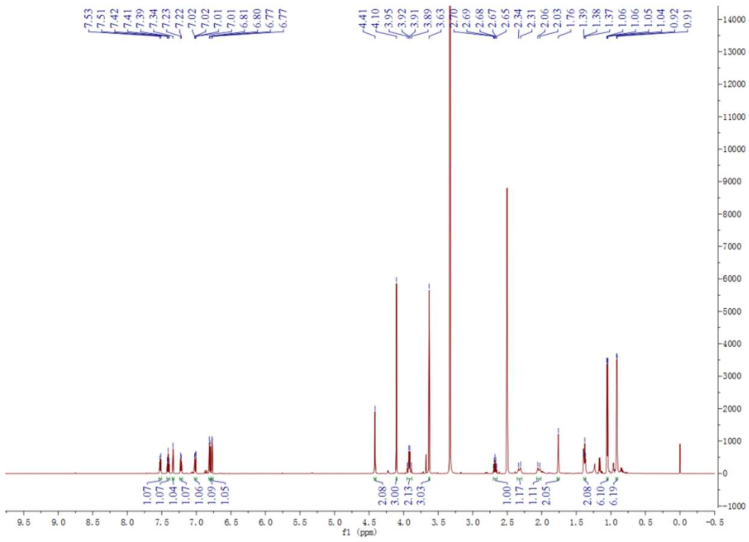

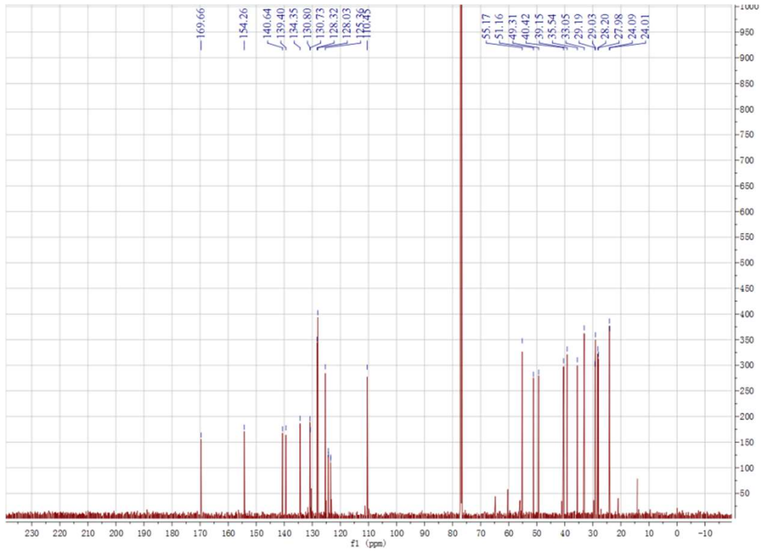

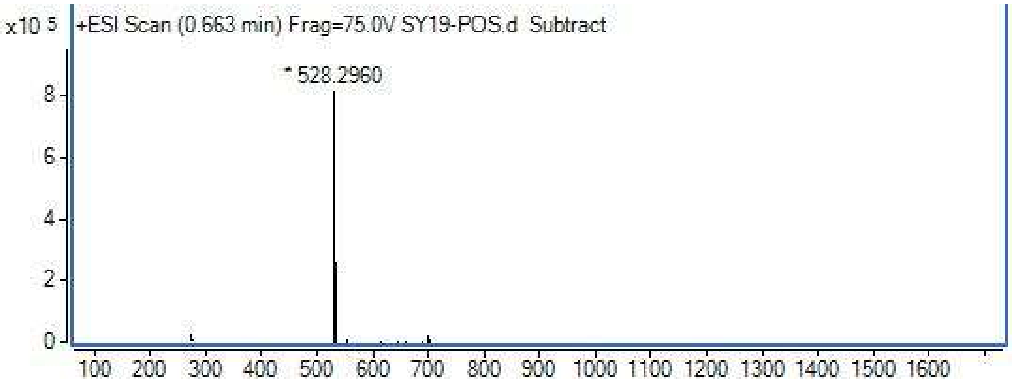
x10 5
+ESI Scan (0.663 min) Frag=75.0V SY19-POS.d Subtract
* 528.2960
8
6
4
2
0
100 200 300 400 500 600 700 800 900 1000 1100 1200 1300 1400 1500 1600

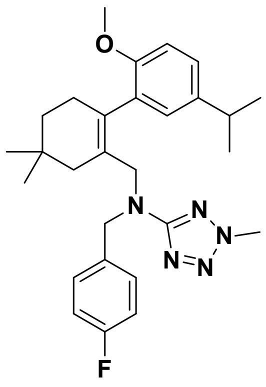
O
N
N
N
N
N
F

Compound 42

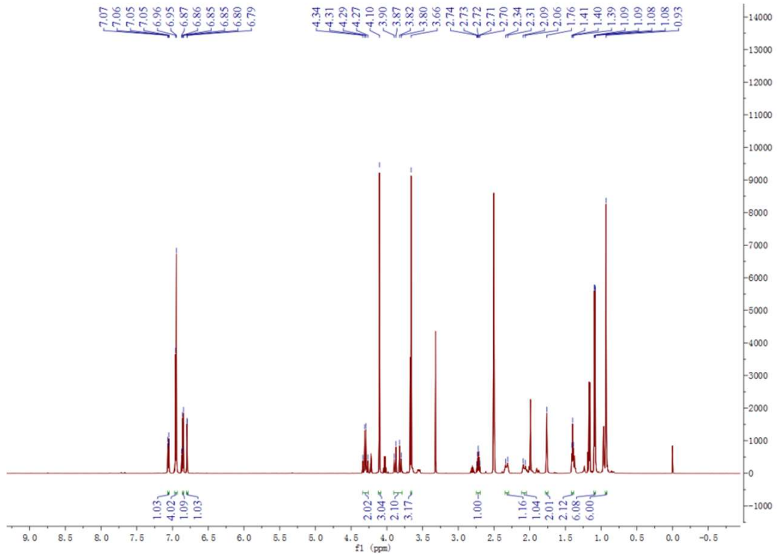
7.07
7.06
7.05
7.05
6.96
6.95
6.87
6.86
6.85
6.85
6.80
6.79
4.34
4.31
4.29
4.27
4.10
3.90
3.87
3.82
3.80
3.66
2.74
2.73
2.72
2.71
2.70
2.34
2.31
2.09
2.06
1.76
1.41
1.40
1.39
1.09
1.09
1.08
1.08
0.93
1.03
4.02
1.09
1.03
2.02
3.04
2.10
3.17
1.00
1.16
1.04
2.01
2.12
6.08
6.00
f1 (ppm)

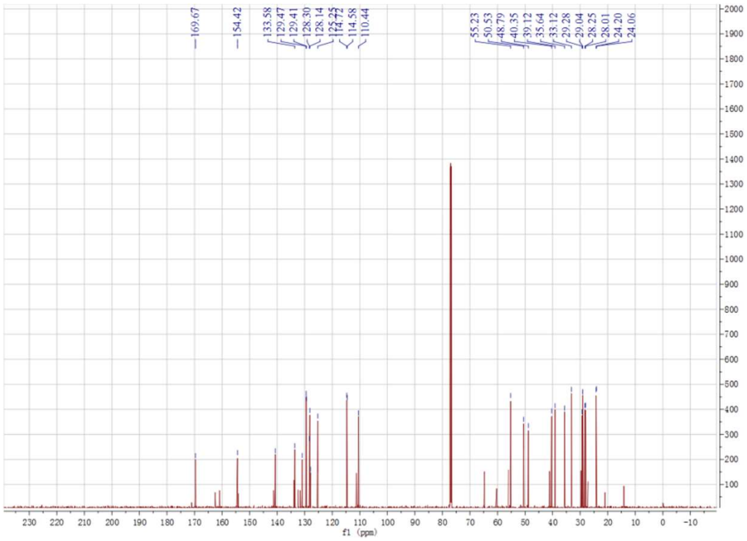
169.67
154.42
133.58
129.47
129.41
128.30
128.14
125.25
114.72
114.58
110.44
55.23
50.53
48.79
40.35
39.12
35.64
33.12
29.28
29.04
28.25
28.01
24.20
24.06
f1 (ppm)

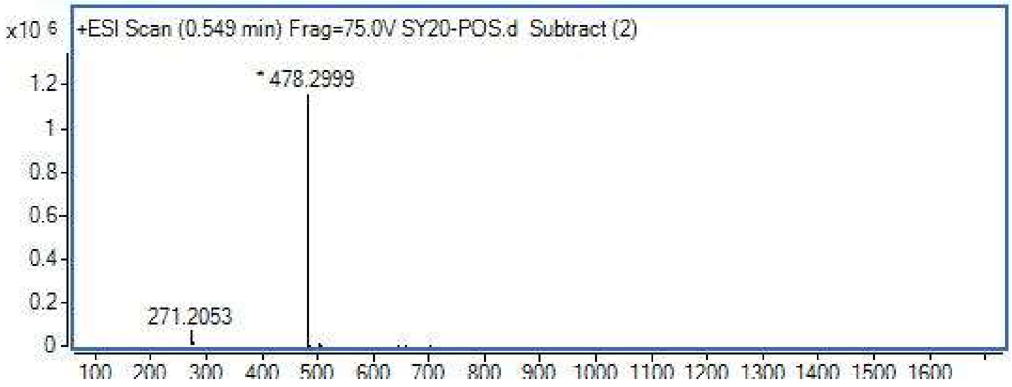
x10 6
+ESI Scan (0.549 min) Frag=75.0V SY20-POS.d Subtract (2)
* 478.2999
271.2053
1.2
1
0.8
0.6
0.4
0.2
0
100 200 300 400 500 600 700 800 900 1000 1100 1200 1300 1400 1500 1600

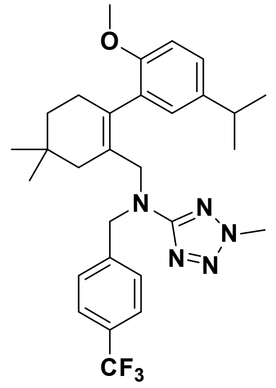

Compound 43

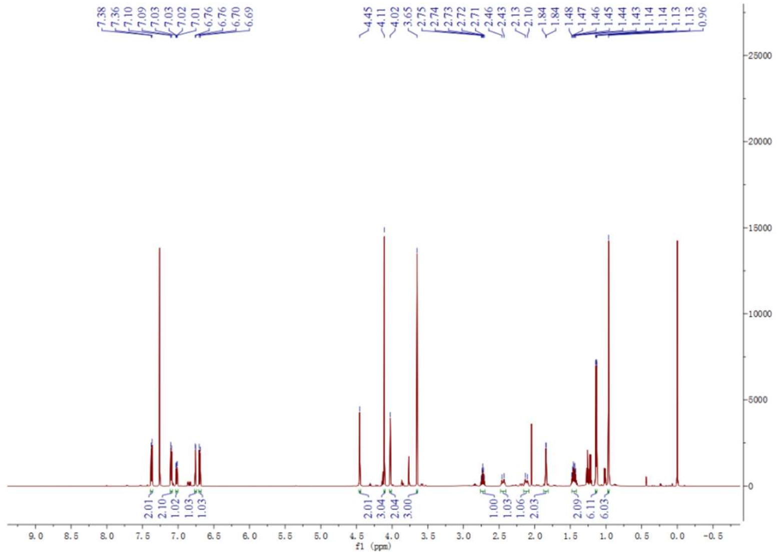

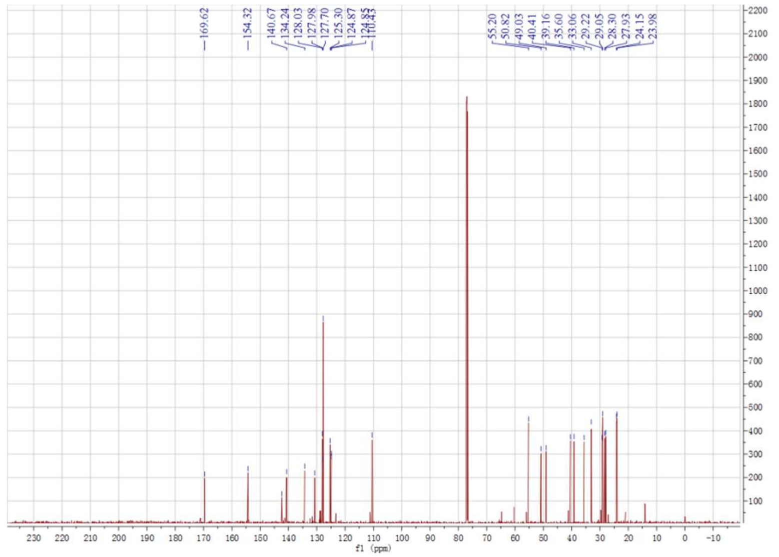

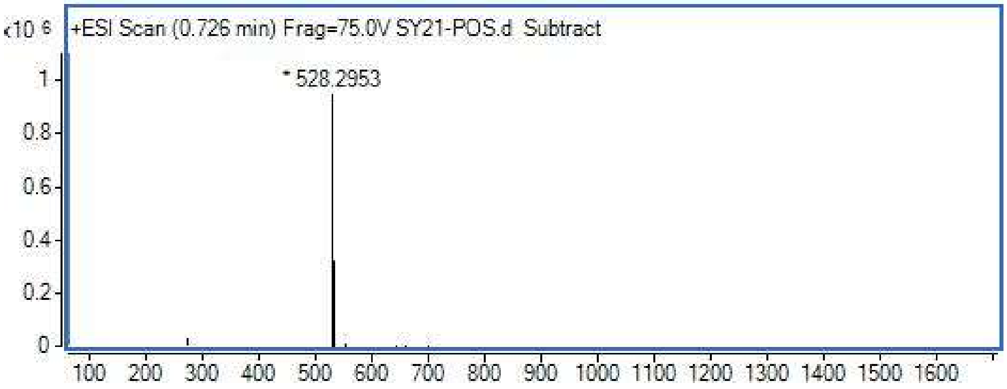

x10 6
+ESI Scan (0.726 min) Frag=75.0V SY21-POS.d Subtract
* 528.2953
1
0.8
0.6
0.4
0.2
0
100 200 300 400 500 600 700 800 900 1000 1100 1200 1300 1400 1500 1600

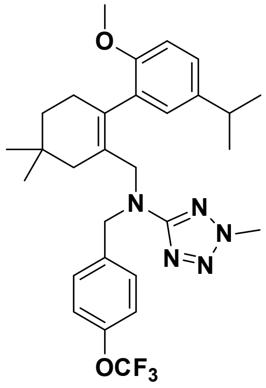

Compound 44

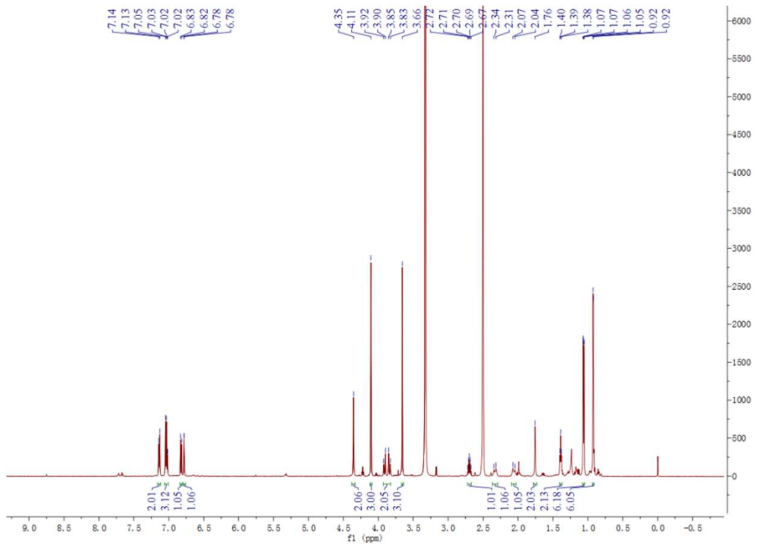

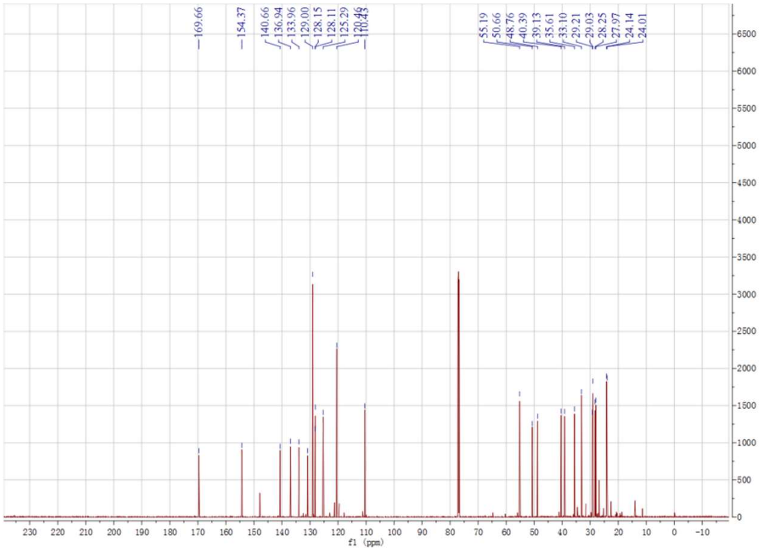

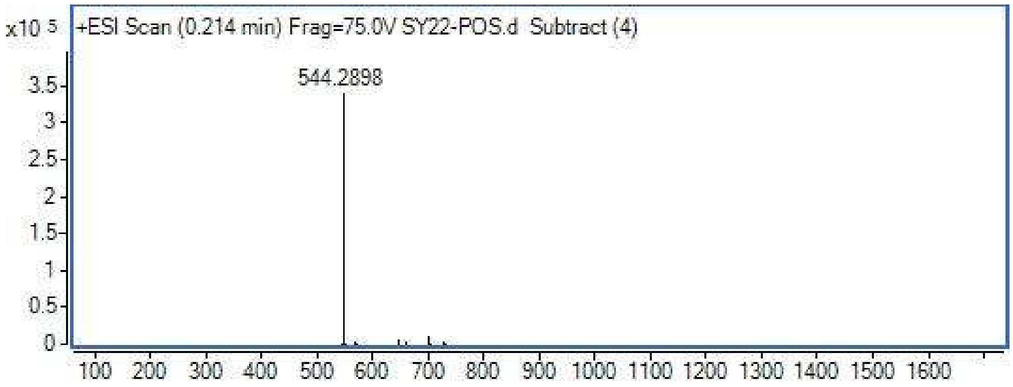

x10 5
+ESI Scan (0.214 min) Frag=75.0V SY22-POS.d Subtract (4)
544.2898
3.5
3
2.5
2
1.5
1
0.5
0
100 200 300 400 500 600 700 800 900 1000 1100 1200 1300 1400 1500 1600
